# Identification of Marker-Trait Associations for Lint Traits in Cotton

**DOI:** 10.3389/fpls.2017.00086

**Published:** 2017-02-06

**Authors:** Muhammad A. Iqbal, Mehboob-ur- Rahman

**Affiliations:** ^1^Plant Genomics and Molecular Breeding Lab (PGMB), Agricultural Biotechnology Division, National Institute for Biotechnology and Genetic Engineering (NIBGE)Faisalabad, Pakistan; ^2^Department of Biotechnology, Pakistan Institute of Engineering and Applied Sciences (PIEAS)Nilore, Islamabad, Pakistan

**Keywords:** association mapping, cotton germplasm, linkage disequilibrium, QTLs, marker-trait associations

## Abstract

Harvesting high quality lint, a long-awaited breeding goal—accomplished partly, can be achieved by identifying DNA markers which could be used for diagnosing cotton plants containing the desired traits. In the present studies, a total of 185 cotton genotypes exhibiting diversity for lint traits were selected from a set of 546 genotypes evaluated for fiber traits in 2009. These genotypes were extensively studied for three consecutive years (2011–2013) at three different locations. Significant genetic variations were found for average boll weight, ginning out turn (GOT), micronaire value, staple length, fiber bundle strength, and uniformity index. IR-NIBGE-3701 showed maximum GOT (43.63%). Clustering of genotypes using Ward's method was found more informative than that of the clusters generated by principal component analysis. A total of 382 SSRs were surveyed on 10 *Gossypium hirsutum* genotypes exhibiting contrasting fiber traits. Out of these, 95 polymorphic SSR primer pairs were then surveyed on 185 genotypes. The gene diversity averaged 0.191 and the polymorphic information content (PIC) averaged 0.175. Unweighted pair group method with arithmetic mean (UPGMA), principal coordinate analysis (PCoA), and STRUCTURE software grouped these genotypes into four major clusters each. Genetic distance within the clusters ranged from 0.0587 to 0.1030. A total of 47 (25.41%) genotypes exhibited shared ancestry. In total 6.8% (*r*^2^ ≥ 0.05) and 4.4% (*r*^2^ ≥ 0.1) of the marker pairs showed significant linkage disequilibrium (LD). A number of marker-trait associations (in total 75) including 13 for average boll weight, 18 for GOT percentage, eight for micronaire value, 18 for staple length, three for fiber bundle strength, and 15 for uniformity index were calculated. Out of these, MGHES-51 was associated with all the traits. Most of the marker-trait associations were novel while few validated the associations reported in the previous studies. High frequency of favorable alleles in cultivated varieties is possibly due to fixation of desirable alleles by domestication. These favorable alleles can be used in marker assisted breeding or for gene cloning using next generation sequencing tools. The present studies would set a stage for harvesting high quality lint without compromising the yield potential—ascertaining natural fiber security.

## Introduction

Cotton, a leading natural textile fiber crop, is comprised of 52 species (Li et al., 2014). Among these, 46 are diploids (2n = 2x = 26) while six are tetraploids including one purported tetraploid species (2n = 4x = 52). Historically, high quantity and quality lint production remained the major goal of almost all breeding research institutes—resulted in marginal success. Major handicapped in improvement of fiber traits is the complex genetics as well as negative correlations occur among quality traits (Zhang and Percy, 2007; Rahman et al., 2014). Many classical procedures, e.g., ANOVA, for testing the significance among the means of various traits were used extensively. New statistical methodologies coupled with a parallel evolution in computational tools paved the way for studying the genetic variability among the cotton genotypes in multiple locations. Principal component analysis (PCA) and cluster analysis are the most robust examples which can provide us comprehensive information about the genotypes understudy. In many experiments, multivariate analysis has been deployed to assess the genetic variability among cotton genotypes (Guan et al., 2012; Zhu et al., 2013).

Narrow genetic base another menace which hinders the breeding progress which has been demonstrated in multiple studies (Rahman et al., 2002, 2007). Also, the narrow genetic base makes the productivity of cotton vulnerable to various insect pests and diseases. Most cultivated varieties possess narrow genetic base because of the fact that breeders usually utilize adapted cotton material for developing new cultivars which result in thinning down the extent of genetic diversity among the cultivated cotton varieties (Van Esbroeck et al., 1999).

Exploitation of molecular quantitative genetics has a great potential to map the quantitative trait loci (QTLs) for yield, fiber quality traits and disease resistance—paved the way to marker assisted selection for genetic improvement.

The extent of the available genetic diversity in cotton has been assessed through means like pedigree and morphological data (May et al., 1995; Van Esbroeck et al., 1999), biochemical markers (Wendel et al., 1992), and DNA based markers (John et al., 2012). At present, genetic diversity estimates are being calculated using different kinds of DNA makers and have been extensively used in cotton e.g., restriction fragment length polymorphism (RFLP; Van Becelaere et al., 2005), amplified fragment length polymorphism (AFLP; Abdalla et al., 2001), random amplified polymorphic DNA (RAPD; Rahman et al., 2007), simple sequence repeat (SSR; Zhang Y. et al., 2011; Tyagi et al., 2014), and inter simple sequence repeat (ISSR; Liu and Wendel, 2001). Among these, SSRs are robust because of the co-dominance nature and high reproducibility rate. These markers have also been used to tag QTLs using the biparental segregating populations (Lacape et al., 2010; Yu et al., 2013) and recombinant inbred lines (Wang et al., 2015). The availability of huge genomic information coupled with the availability of the various high-tech statistical and bioinformatics tools have laid the foundation for understanding the extent of genetic diversity, development of genetic maps, understanding QTLs, and their effects (Jiao et al., 2010).

In multiple investigations, model based structured analysis have been extensively used to determine the population structure (Tyagi et al., 2014; Zhao et al., 2014). Association mapping or LD mapping is an alternative technique for mapping the QTLs and estimating their effect. In this type of analysis, genetically diverse germplasm is used than that of the biparental linkage mapping (Luo et al., 2008). LD mapping exploits the nonrandom association of loci present in various genotypes to dissect the complex traits by exploring large number of historical recombination events occurred in the natural population (Ersoz et al., 2007). Moreover, association mapping detects the polymorphism(s) within a gene confers a trait (Yan et al., 2010). Cotton has been studied for the prospects and application of association mapping for fiber related traits (Zeng et al., 2009; Cai et al., 2014; Nie et al., 2016), yield and its components (Zhang et al., 2013), salinity tolerance (Saeed M. et al., 2014), agronomic traits (Kalivas et al., 2011), drought and salt tolerance (Jia et al., 2014; Zhao et al., 2016), plant structure (Li et al., 2016b), early maturing traits (Li et al., 2016a), and seed oil and protein contents (Liu et al., 2015).

Different marker systems, marker types, and population under study have made it virtual to compare the identified QTLs in different laboratories. Complex nature of fiber related traits and major role of G X E within different generation and locations is a bottleneck for the repeatability of the QTLs. However, some identified QTLs across different laboratories are reproducible. High-through genotyping arrays have numerous applications like high density genetic mapping, genome-wide association studies (GWAS), genomic selection (GS), dissection of quantitative traits, and studying the pattern of genetic diversity among the genotypes and their wild relatives. A study was conducted to develop the CottonSNP63K, an Illumina Infinium array containing assays for 45,104 putative intraspecific single nucleotide polymorphism (SNP) markers for use within the cultivated cotton species *Gossypium hirsutum* L. and 17,954 putative interspecific SNP markers for use with crosses of other cotton species with *G. hirsutum*. The array was validated with 1156 samples to generate cluster positions to facilitate automated analysis of 38,822 polymorphic markers (Hulse-Kemp et al., 2015).

This study would be novel (germplasm from different countries, Pakistani germplasm, also tested in Pakistani environment where temp is high). Thus, the following studies were planned for studying the expression of various fiber traits of the available cotton germplasm, estimation of genetic diversity among these genotypes and to measure the genomic distribution of linkage disequilibrium (LD) between SSRs and the traits understudy.

## Materials and methods

The present study was conducted in Plant Genomic & Molecular Breeding Lab (PGMB), Agricultural Biotechnology Division (ABD), National Institute for Biotechnology and Genetic Engineering (NIBGE), Faisalabad affiliated with Pakistan Institute of Engineering and Applied Sciences (PIEAS), Nilore Islamabad.

### Plant material

Plant material consisted of 546 cotton genotypes belonging to tetraploid as well as diploid cotton species e.g., *G. hirsutum* and *Gossypium arboreum*. These genotypes were sown at NIBGE Faisalabad in 2009. A total of 50 bolls from each genotype were picked. The seed cotton was ginned and fiber traits were measured through HVI-1000. Genotypes exhibiting same staple length were discarded and the rest of the genotypes were divided into three fiber groups (small, medium, and large). In total, 185 cotton genotypes were selected for detailed studies which were representative of exotic, local *Bt*-cotton and desi (*G. arboreum*) genotypes. The genotypes of *G. arboreum* were originated and bred in Pakistan except few genotypes. Hereafter, all accessions/genotypes/cultivars/varieties will be referred as genotypes in the whole manuscript. Names of the selected 185 cotton genotypes along with their genome, chromosome number and parentage/accession number are given in Supplementary Table 1.

These genotypes were grown for three consecutive years (2011–2013), at three distinct locations of major cotton growing districts of Pakistan (Faisalabad, Vehari, and Multan) to study lint traits. Uniform piece of land was used for growing the cotton genotypes to avoid experimental errors. Alpha-lattice design was used to conduct this experiment. At each location, the trial was laid out with two replications of 37 incomplete blocks consisting of five entries (genotypes) in each block. The randomization was done by alpha program while the net plot size of experiment for each entry (genotype) in each replication was 21 m^2^ (4 rows 7 m long with spacing of 0.75 m). The sowing was completed from May 20 to 27 each year by hand drill method. The plant-to-plant distance was kept 30 cm apart with in a row. Adequate number of irrigations was applied for avoiding water stress, especially during reproductive stage. Every year, the cotton crop was supplemented with 50 kg ha^−1^ of NPK as a pre-planting application followed by 50 kg ha^−1^ of N application each at flowering and boll setting stage. Similarly, chemical control measures were taken to protect cotton crop from sucking and chewing (bollworm complex) insect pests. Uniform agronomic practices were applied at each site during the 3 consecutive years (2011–2013).

### Measurement of fiber quality traits

A total of 50 bolls were picked randomly from all positions of cotton plants from the central two rows of each genotype grown at three different locations (NIBGE Faisalabad, CRS Vehari, and CCRI Multan) for three consecutive cotton growing seasons 2011–2013. After weighing the total weight of the 50 bolls, the average boll weight was calculated by dividing the total weight of seed cotton with the total number of bolls (50 bolls). The samples were ginned on small saw-gin machine. Lint weight and seed weight were measured on electrical balance and ginning out turn (GOT) percentage was calculated by the following formula:

$$\begin{array}{l} \text{GOT }\%\;=\text{Lint weight}/\text{Total seed cotton weight}{\;}^{*}\;100 \end{array}$$

Fiber quality traits (micronaire value, staple length, fiber bundle strength, and uniformity index) were measured on High Volume Instrument (HVI) model USTER® HVI 1000 at the PGMB lab, NIBGE Faisalabad. For fiber analysis through HVI, 50 g of cotton lint sample was used. Fiber quality traits were tested at 20°C and 65% relative humidity.

The summary statistics for all the studied traits was calculated for the three normal cotton growing seasons for each of the three locations with statistix 8.1 and frequency distribution of all the six traits was calculated through pivot table. Analysis of variance (ANOVA) appropriate for the specified experimental design was performed using alpha lattice design with alpha lattice software for experiments conducted to determine the significance of difference among genotypes for the three locations (NIBGE Faisalabad, CRS Vehari, and CCRI Multan) for three cotton growing seasons 2011–2013. Statistical significance was assumed at 5 and 1% levels of probability. Correlation analysis was performed with the help of a software (SPSS 16) for estimating the relationships among various traits. Statistica software was used for principal component analysis and cluster analysis to find the relationship among 185 cotton genotypes.

### SSR genotyping

Total genomic DNA of all the cotton genotypes was extracted from 2 to 3 leaves excised from each genotype. The leaves were washed with distilled water to remove dust and any other foreign particles. The leaf samples were placed in small plastic bags, carried immediately to the laboratory and stored at −80°C until used for DNA extraction. Total genomic DNA was extracted using a modified CTAB method (Iqbal et al., 1997). Quantity of the genomic DNA was estimated using nanodrop. The quality and quantity was further confirmed by running 50 ηg of genomic DNA of each genotype on 0.8% agarose gel. Dilutions were made from the extracted DNA stocks. The stock solutions and dilutions were stored at −20°C.

Initially, a total of 382 SSR primer pairs (42 MGHES, 316 PR, five PR-GR-BES, 16 BNL, two JESPR, and one from CM series) were surveyed on 10 selected cotton genotypes (five each from short staple as well as long staple length group). Sequences of few primers (61) were taken from the published data while 321 primer pairs were newly synthesized SSRs, designated as PR and PR-GR-BES—designed from the sequences containing repeats in the BAC end cloned sequences (available at the Plant Genome Mapping Laboratory, University of Georgia, Athens, USA) of *G. raimondii* genome sequences. For this purpose PRIMER-3 software was used and the primers containing >40% GC contents were picked. Primers were custom synthesized from Eurofins Genomics, Ebersberg, Germany. A total of 95 SSR primer pairs were found polymorphic which were further surveyed on 185 cotton genotypes to find the polymorphic loci.

The PCR conditions (annealing temperature etc.) were optimized for the newly designed SSRs. PCR volume was 20 μl and reaction mixture contained 30 ηg DNA, 2.5 mM dNTPs, 30 ηg forward and reverse primer each, 1 unit of *Taq* DNA polymerase with 10X reaction buffer and 25 mM MgCl_2_. All primers were amplified in a thermal cycler (Eppendorf, made in Germany) using various temperature regimes like 94°C for 5 min (one cycle), 35 cycles each of 94°C for 30 s, 50–60°C (depending on the annealing temperature of each primer) for 30 s, 72°C for 1 min. Finally the PCR tubes were heated at 72°C for 5 min. After PCR amplification, the amplicons were resolved on agarose as well as on metaPhor™ (Cambrex Corporation, USA) agarose. For this purpose 3 μL of gel loading dye (Bromophenol blue) was added directly to the reaction tubes and spun for few seconds in a micro centrifuge after mixing with the entire reaction mixtures. Gel images captured through gel documentation system were placed in the Microsoft excel sheet with genotypes names. The amplicons were scored (1 for present while 0 for absent).

For each SSR, number of amplicons, polymorphic alleles, numbers of observed, and effective alleles were calculated. Basic statistics summary including major allele frequency, gene diversity, heterozygosity, and allelic diversity was calculated with the help of a Power Marker version 3.25 (Liu and Muse, 2005). Polymorphic information content (PIC)-values for each SSR were calculated as described by Botstein et al. (1980) through the following formula:

$$\begin{array}{l} \text{PI}{\text{C}}_{j}=1-\overset{n}{\underset{i=1}{\sum }}pi^{2} \end{array}$$

For the phylogenetic tree, genetic distances were calculated using previously described formula (Nei et al., 1983). These genetic distances were used to construct a phylogeny using unweighted group pair method of arithmetic mean (UPGMA). A Power Marker software was used for tree construction and tree was viewed with the help of tree view software. NTSYSpc version 2.10t software was deployed to perform PCoA.

### Population structure

Population structure of 185 cotton genotypes was estimated using a STRUCTURE software V2.2 based on Bayesian clustering algorithm (Pritchard et al., 2000; Falush et al., 2003). In order to conclude the optimum number of subpopulations, values for *K* = 2 to *K* = 10 were given using a burn length of 20,000 and run length of 20,000. Five independent runs yielded the reproducible results. The results were imported to STRUCTURE HARVESTER software to calculate exact value of ΔK (Earl and Vonholdt, 2012).

### Linkage disequilibrium (LD)

The level of LD and its significance for SSR marker pair loci was calculated (*P* < 0.0001) using the TASSEL software package. LD was estimated for the 185 cotton genotypes. The analysis was conducted both with and without consideration of the admixed genotype identified by structure at *K* = 4. The LD was estimated by computing the squared correlation coefficient (*r*^2^). The LD-values between all pairs of SSR loci were plotted as triangle LD plots to estimate the general view of genome-wide LD patterns and evaluate “block-like” LD structures.

### Association mapping

The software program TASSEL 2.01 was used to calculate associations between SSRs and trait. Two models such as a general linear model (GLM) based on Q-matrix derived from STRUCTURE and a mixed linear model (MLM; Yu and Buckler, 2006) based on Q-matrix and the kinship-matrix, were deployed for calculating the associations between the marker and the trait. In association analysis, *P*-value describes the association between markers and QTLs while *r*^2^ describes the effects of QTLs. Significance level for *P*-value was set at 0.0001(LOD = 3) for all traits except fiber strength for which LOD was set at 2.

## Results

### Mean performance of cotton genotypes

A total of 185 cotton genotypes (out of 546) were selected for conducting detailed analysis of a number of traits including average boll weight, GOT percentage and fiber quality. These trials were conducted on three locations for 3 consecutive years. The average boll weight of three locations for 3 years was 2.73 g (ranging from 1.14 g for “Garohill” to 3.67 g for “DT-Webber”). The cumulative mean of GOT percentage was 33.71% (ranging from 17.91% for 23,718 and 43.63% for IR-NIBGE-3701) at all locations for 3 consecutive years (2011–2013). At the three locations from 2011 to 2013, the micronaire value was found in the range of 3.34 to 6.20 μg/inch, having a mean value of 4.93 μg/inch. The staple length (UHML) is one the most important parameters which determines the lint quality. Over all, at the three locations for the three consecutive seasons (2011–2013), lint of cotton genotypes showed a mean staple length of 25.47 mm, ranging from 13.39 mm (DC-116) to 30.22 mm (PAN-F3-5575), followed by 30.17 mm (PGMB-3300). Over all, at the three locations (NIBGE Faisalabad, CRS Vehari, and CCRI Multan) for three consecutive years, the average mean value of fiber bundle strength was shown to be 27.67 g/tex—ranged from 25.00 g/tex (Belonga-5531) to 30.54 g/tex (G.S/LB-602), followed by 30.40 g/tex (1408). Uniformity index (UI), another important parameter of defining fiber quality, was also explored on the available cotton genotypes. The cumulative mean at all the three locations for 3 years (2011–2013) was 79.66%. The UI-values were in the range of 77.22% (for Garohill) to 82.74% (for LB-546).

**Figure 1:**
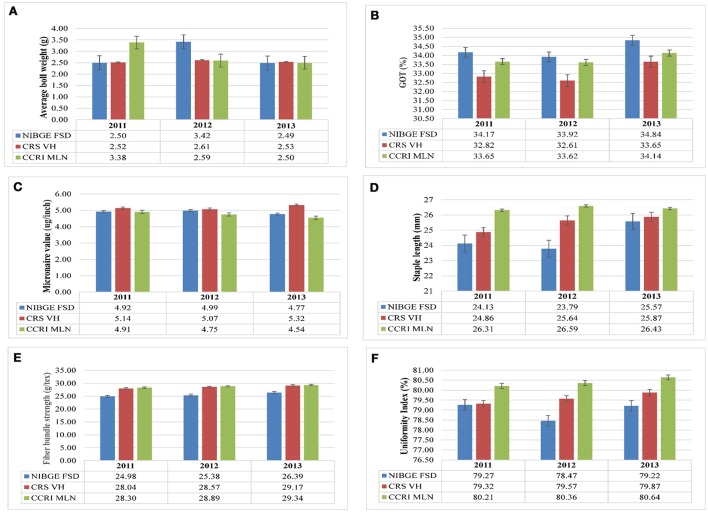
**Comparison of different parameters**. **(A)** average boll weight, **(B)** micronaire value, **(C)** GOT percentage, **(D)** staple length, **(E)** fiber bundle strength and, **(F)** uniformity index of lint samples collected from NIBGE Faisalabad, CRS Vehari and CCRI Multan from 2011 to 2013.

Comparison of average boll weight, GOT, micronaire value, staple length, fiber bundle strength, and uniformity index at NIBGE Faisalabad, CRS Vehari, and CCRI Multan from 2011 to 2013 is given in Figure 1 and the frequency distribution for all the traits is presented in Supplementary Figure 1. Summary statistics for all these studied traits (mean, minimum, and maximum value, S.D, S.E, C.V, and C.D) is given in Supplementary Tables 2–4. The statistical analysis revealed that 185 genotypes included in the study had shown significant variations (*p* ≤ 0.01) for all the traits. The results are summarized in Table 1. Significant trait correlations were observed among all the traits understudy and the results have been given in Table 2.

**Table 1:** **Mean squares of the average boll weight, GOT percentage, micronaire value, staple length, fiber bundle strength and uniformity index at NIBGE Faisalabad, CRS Vehari and CCRI Multan from 2011 to 2013**.

**Locations**	**S.O.V**	**df**	**Average boll weight (g)**	**GOT percentage**	**Micronaire value (μg/inch)**	**Staple length (mm)**	**Fiber bundle strength (g/tex)**	**Uniformity index (%)**
NIBGE Faisalabad	Replications	1	0.0000	1.2607^**^	0.0000	1.9801	0.0005^**^	10.3992^**^
	Blocks	72	0.0181	0.0719	0.0504	0.0496	0.0319	0.2926
	Genotypes	184	0.8703^**^	54.2209^**^	0.4923^**^	26.1963^**^	9.8566^**^	4.9197^**^
	Genotypes (Adjusted)	184	0.4504^**^	31.5055^**^	0.2971^**^	12.9528^**^	4.8978^**^	3.0386^**^
	Error	112	0.0062	27.0782	0.0134	0.0355	0.0086	0.2563
CRS Vehari	Replications	1	0.0003	2.9046^**^	0.0003*ns*	0.0017	0.0001	8.0791^**^
	Blocks	72	0.0155	0.1308	0.0461	0.0505	0.0184	0.3952
	Genotypes	184	0.4967^**^	46.8275^**^	0.5024^**^	29.5880^**^	4.8648^**^	2.2722^**^
	Genotypes (Adjusted)	184	0.2790^**^	26.4989^**^	0.3661^**^	3.7429^**^	2.8778^**^	1.4565^**^
	Error	112	0.0030	0.0838	0.0125	0.0106	0.0051	0.3545
CCRI Multan	Replications	1	0.0001*ns*	0.6714^*^	0.0000	0.0001	0.0004	18.2715^**^
	Blocks	72	0.0115	0.1214	0.0478	0.0101	0.0160	0.3975
	Genotypes	184	0.3986^**^	44.3168^**^	0.6822^**^	35.3844^**^	3.1405^**^	3.5902^**^
	Genotypes (Adjusted)	184	0.1582^**^	23.4556^**^	0.4340^**^	5.9483^**^	1.2984^**^	2.2633^**^
	Error	112	0.0021	0.01016	0.0144	0.0033	0.0056	0.4001

**Table 2:** **Estimates of phenotypic correlation coefficients among average boll weight, GOT percentage, micronaire value, staple length, fiber bundle strength, and uniformity index among 185 cotton genotypes**.

	**Average boll weight**	**GOT percentage**	**Micronaire value**	**Staple length**	**Fiber bundle strength**	**Uniformity index**
Average boll weight	1					
GOT percentage	0.504^**^	1				
Micronaire value	−0.143	−0.153^*^	1			
Staple length	0.552^**^	0.783^**^	−0.315^**^	1		
Fiber bundle strength	0.200^**^	0.318^**^	−0.028	0.426^**^	1	
Uniformity index	0.415^**^	0.528^**^	−0.015	0.608^**^	670^**^	1

** *Correlation is significant at the 0.01 level*,

* *Correlation is significant at the 0.05 level*.

### Principal component analysis (PCA)

**Figure 2:**
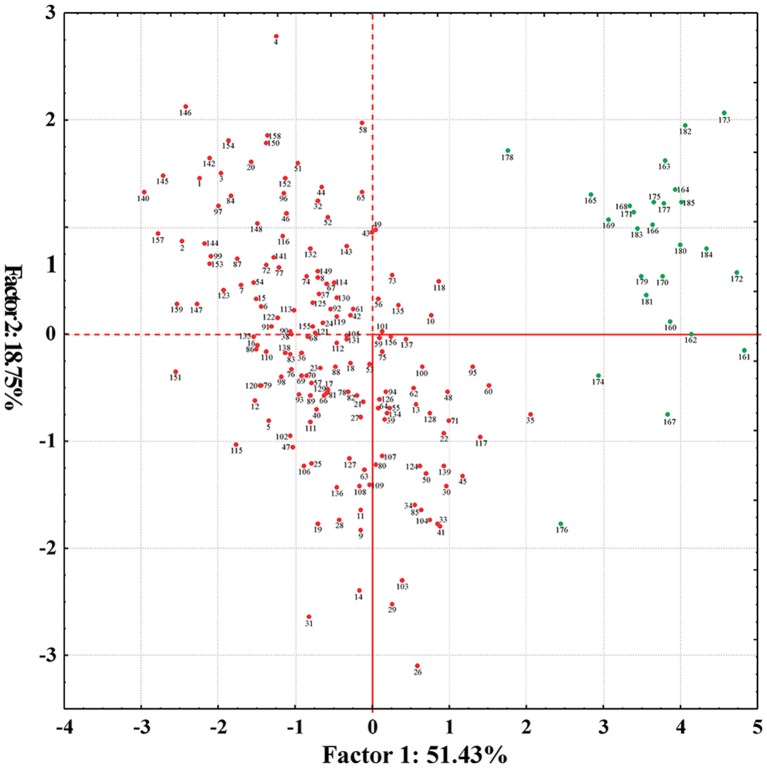
**Principal component analysis scatter plot depicting the genetic diversity based on six phenotypic traits (average boll weight, GOT percentage, micronaire value, staple length, fiber bundle strength, and uniformity index) of 185 cotton genotypes**. Green dots representing the *G. arboreum* genotypes and red dots exhibiting the *G. hirsutum* genotypes.

PCA was carried out by considering all variables simultaneously. The eigen-values indicated the variance of principal components. A total of five principal components (PC1 to PC5) accounted for 97% of the total variation (Supplementary Table 5). The PC1 was comprised of 51%, while PC2, PC3, PC4, and PC5 accounted for 19, 14, 9, and 4% of the total variation, respectively. Characteristics of each principal component were determined on the basis of estimated factor loadings. PC1 was related to average boll weight, GOT percentage, staple length, fiber bundle strength, and uniformity index; PC2 to micronaire value and fiber bundle strength; PC3 to average boll weight, micronaire value, and fiber bundle strength; PC4 to average boll weight and GOT and PC5 to uniformity index. The genotypes of *G. arboreum* and genotypes of *G. hirsutum* were clustered into two groups. However, no evident and significant groups within a species were observed (Figure 2).

### Cluster analysis

**Figure 3:**
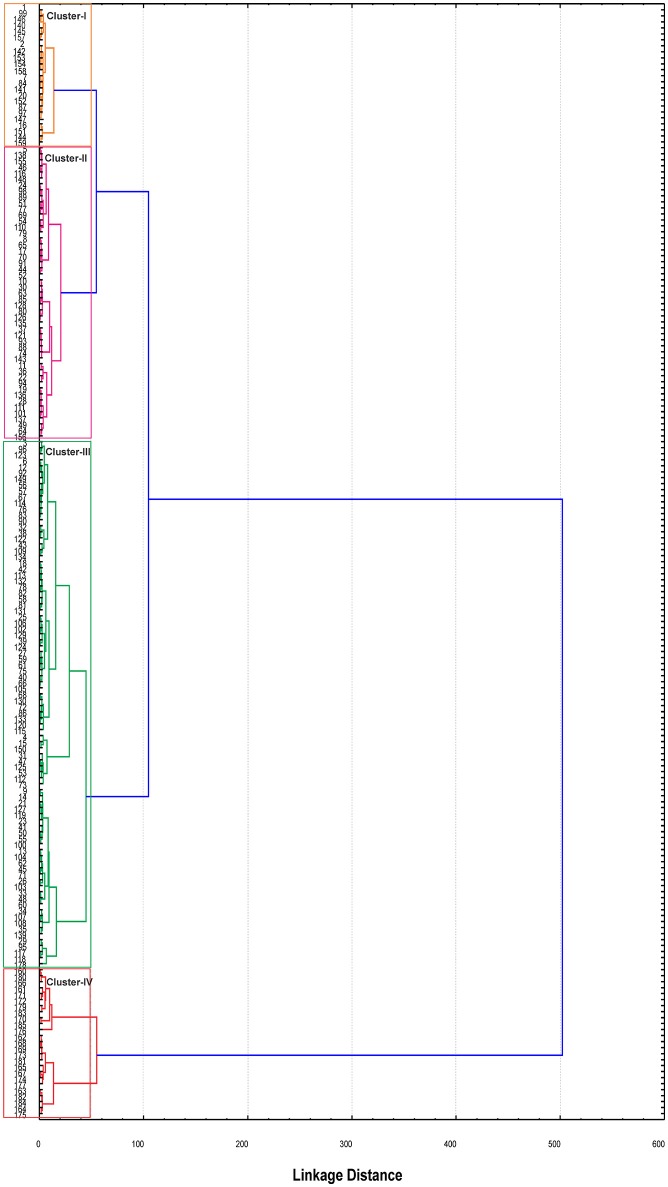
**Dendrogram constructed using Ward's method of cluster analysis based on phenotypic variation among the 185 cotton genotypes**. Colors in the dendrogram correspond to different clusters.

To overcome the concern of grouping of genotypes within same species, Ward's method was deployed to establish relationship among the 185 cotton genotypes. Similar type of genotypes were clustered according to minimal distance analysis on the basis of mean values of the five principal components. All these genotypes were grouped into four clusters. Maximum number of genotypes i.e., 87 were clustered in cluster-III, whereas the minimum number of genotypes (23) were grouped in cluster-I. Cluster-II and IV, comprised of 49 and 26 genotypes, respectively (Figure 3). Cluster mean and general mean for each of the trait are shown in Table 3.

**Table 3:** **Cluster mean and general mean for different phenotypic traits in cotton**.

**Sr. no**.	**Trait/Cluster**	**I**	**II**	**III**	**IV**	**General mean**
1	Average boll weight (g)	2.84	2.86	2.83	2.02	2.73
2	GOT percentage	38.05	36.75	33.81	23.84	33.71
3	Micronaire value (μg/inch)	5.00	4.98	4.78	5.29	4.93
4	Staple length (mm)	28.43	26.35	26.77	16.80	25.47
5	Fiber bundle strength (g/tex)	29.39	27.43	27.63	26.74	27.67
6	Uniformity index (%)	81.38	79.78	79.59	78.13	79.66

### Assessment of genetic diversity among cotton genotypes

A total of 10 genotypes of *G. hirsutum* panel showing extreme variation in the staple length, were selected for preliminary screening with SSR primers. In total, 382 SSR primer pairs were surveyed on 10 cotton genotypes. A total of 95 primer pairs were found polymorphic—showing 25.74% polymorphism. However, 13 primer pairs did not amplify. The polymorphic SSRs were then surveyed on the genomic DNA of 185 cotton genotypes. The number of amplified alleles per SSR primer pair varied from 01 to 06 with an average of 1.71 alleles/marker. A total 162 alleles were amplified by these polymorphic SSRs, out of these 144 (88.88%) alleles were polymorphic. A total of 56 SSRs produced single allele, 20 SSRs produced two alleles, 13 SSRs produced three alleles, four SSRs yielded four alleles while five and six alleles were produced by single SSR each. The primer MGHES-60 amplified the maximum number of polymorphic alleles (5). A total of 68 SSR primer pairs amplified the minimum number of polymorphic alleles (1) (Table 4).

**Table 4:** **Total number of amplified alleles, and their status (polymorphic/monomorphic) of 95 SSR primer pairs**.

**Sr no**.	**Markers name**	**Allele no**.	**Polymorphic**
1	MGHES-3	3	3
2	MGHES-4	1	1
3	MGHES-5	2	2
4	MGHES-6	2	2
5	MGHES-7	1	1
6	MGHES-12	1	1
7	MGHES-13	1	1
8	MGHES-14	5	5
9	MGHES-15	2	2
10	MGHES-16	1	1
11	MGHES-18	3	3
12	MGHES-19	1	1
13	MGHES-20	2	1
14	MGHES-21	2	1
15	MGHES-22	2	2
16	MGHES-23	2	2
17	MGHES-26	1	1
18	MGHES-28	1	1
19	MGHES-31	1	1
20	MGHES-32	1	1
21	MGHES-33	1	1
22	MGHES-34	2	2
23	MGHES-36	4	4
24	MGHES-37	2	2
25	MGHES-39	1	1
26	MGHES-41	1	1
27	MGHES-42	3	3
28	MGHES-43a	1	1
29	MGHES-45	1	1
30	MGHES-46	1	1
31	MGHES-47	1	1
32	MGHES-48	1	1
33	MGHES-49	1	1
34	MGHES-50	1	1
35	MGHES-51	2	1
36	MGHES-52	1	1
37	MGHES-53	2	1
38	MGHES-55	2	1
39	MGHES-60	6	5
40	MGHES-63	3	1
41	MGHES-67	3	2
42	MGHES-72	1	1
43	MGHES-75	3	3
44	MGHES-78	1	1
45	PR-GR-BES-2	1	1
46	PR-GR-BES-4	3	2
47	PR-GR-BES-5	1	1
48	PR-GR-BES-6	1	1
49	PR-GR-BES-7	1	1
50	PR-1	1	1
51	PR-2	2	1
52	PR-3	1	1
53	PR-4	1	1
54	PR-5	1	1
55	PR-6	1	1
56	PR-7	1	1
57	PR-8	3	3
58	PR-11	1	1
59	PR-13	1	1
60	PR-16	2	1
61	PR-18	2	1
62	PR-19	3	1
63	PR-21	2	1
64	PR-22	1	1
65	PR-25	4	4
66	PR-67	1	1
67	PR-69	2	2
68	PR-70	2	1
69	PR-82	3	2
70	PR-91	3	3
71	PR-162	3	3
72	Pr-193	1	1
73	PR-195	1	1
74	PR-231	1	1
75	PR-237	1	1
76	PR-471	1	1
77	JESPR295	1	1
78	JESPR307	1	1
79	BNL569	1	1
80	BNL-1122	1	1
81	BNL-1227	4	4
82	BNL-1604	1	1
83	BNL-1667	1	1
84	BNL-1672	3	3
85	BNL-2921	1	1
86	BNL-245	1	1
87	BNL-2960	1	1
88	BNL-3071	1	1
89	BNL-3090	1	1
90	BNL-3408	4	4
91	BNL-3410	1	1
92	BNL-3445	1	1
93	BNL-3569	2	2
94	BNL-4017	1	1
95	CM-43	2	2
	Total	162	144 (88%)

**Figure 4:**
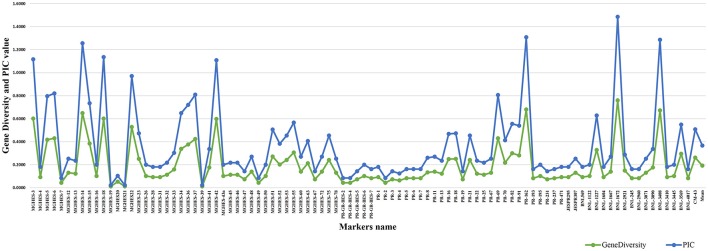
**Histogram showing the gene diversity and PIC-value against SSR markers**.

The major allele frequency varied from 0.373 (BNL-1672) to 1.000 (MGHES-19) with an average of 0.877 (Supplementary Figure 2). The PIC-value was calculated to determine the level of polymorphism and informativeness of each SSR primer pair. The highest PIC-value was calculated for the primer BNL-1672 (0.726) while the lowest was found for MGHES-19 (0.010) with an average of 0.175. However, the range of gene diversity was found to be 0.010 (MGHES-19) to 0.760 (BNL-1672) with a mean value of 0.191 (Figure 4). A total of 14 species-specific markers, viz. MGHES-14, MGHES-15, MGHES-18, MGHES-23 MGHES-34, MGHES-36, MGHES-37, MGHES-42, MGHES-53, MGHES-75, PR-18, PR-21, BNL-1227, and BNL-3569 were identified which can distinguish between *G. arboreum* and *G. hirsutum*.

### Genetic distance and cluster analysis

**Figure 5:**
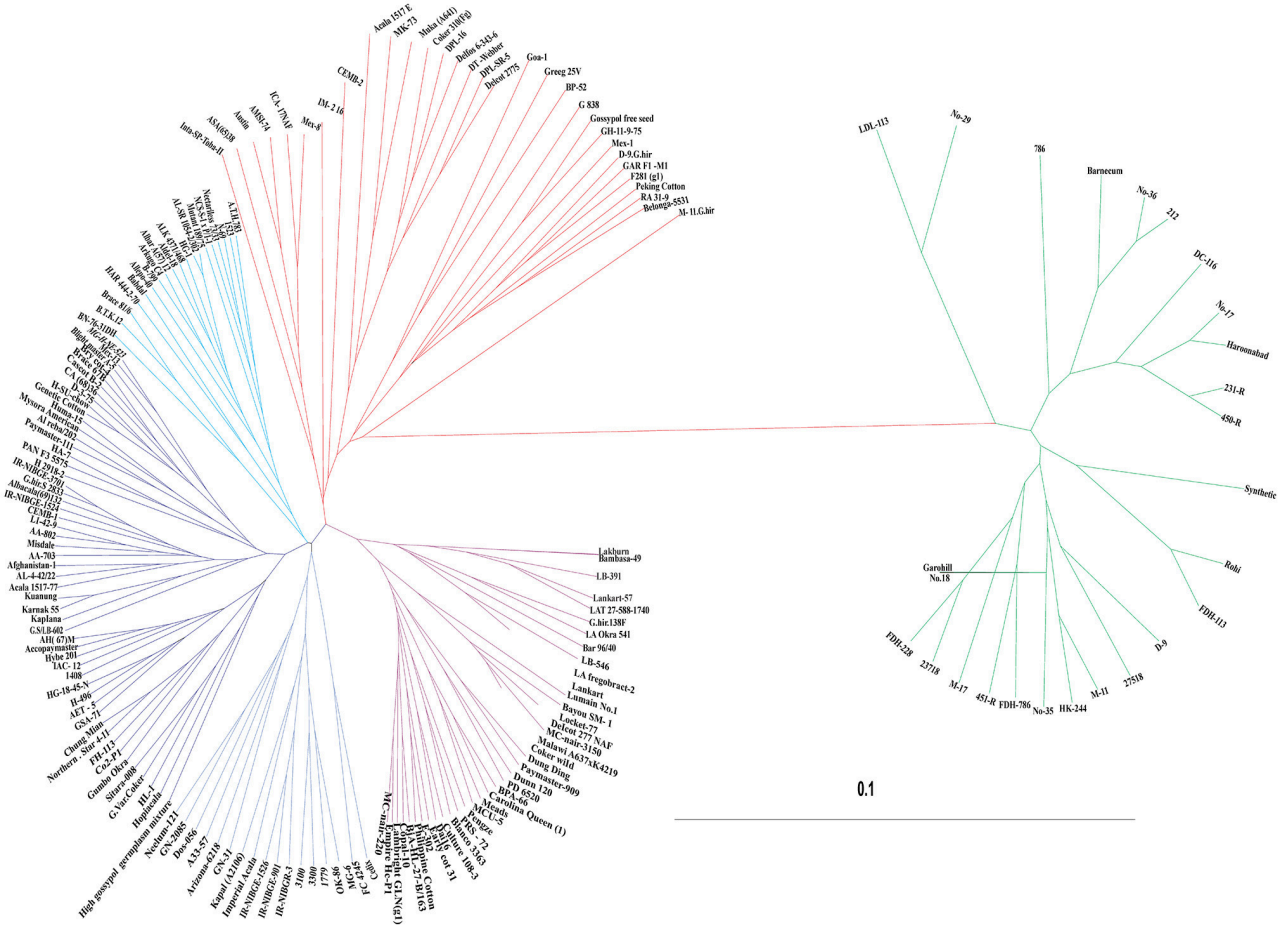
**UPGMA tree constructed using the dissimilarity matrix of 185 cotton genotypes**. Colors in the dendrogram correspond to different clusters; Cluster-I (Green), Cluster-II (Red), Cluster-III (Blue), and Cluster-IV (Purple).

Genetic dissimilarity (diversity) coefficients ranged from 0.003 to 0.235 with an average of 0.098. Minimum dissimilarity coefficient (0.003) was observed between “AL-SR-1054-2/302” and “HG-1.” While maximum dissimilarity coefficient (0.235) was observed between “Barnecum” and “Peking cotton.” Among the 185 genotypes, “DC-116” was found the most diverse genotype—its average dissimilarity was 0.163. In total, four clusters were generated. All genotypes (in total 26) representing *G. arboreum* were grouped in one cluster while the *G. hirsutum* genotypes were grouped in remaining three clusters (Figure 5). For example, Cluster-II consisted of 31 genotypes including one *Bt*-cotton genotype “CEMB-2.” Cluster-III was comprised of 89 genotypes which was further subdivided into three sub clusters, IIIA, IIIB, and IIIC. Sub cluster-IIIA had 19 genotypes including one *Bt*-cotton genotype “PGMB-1523.” Sub cluster-IIIB comprised of 52 genotypes including six *Bt*-cotton genotypes; “IR-NIBGE-3701,” “IR-NIBGE-1524,” “CEMB-1,” “AA-802,” “AA-703,” and “Sitara 008,” while most of the *Bt*-cotton genotypes were assigned to sub cluster-IIIC. Cluster-IV contained 39 genotypes (mostly exotic genotypes). Minimum genetic distance (0.0587) was observed within cluster-IV showing less diversity followed by cluster-I (0.0588) and then cluster-III (0.0659). Maximum genetic distance (0.1030) was observed within cluster-II, indicating the presence of diverse genotypes. In comparison between the clusters, maximum genetic distance (0.1723) was observed between cluster-I and cluster-II followed by the genetic distance (0.1694) between cluster-I and IV. Minimum genetic distance (0.0779) was observed between cluster-III and IV.

### Principle coordinate analysis

**Figure 6:**
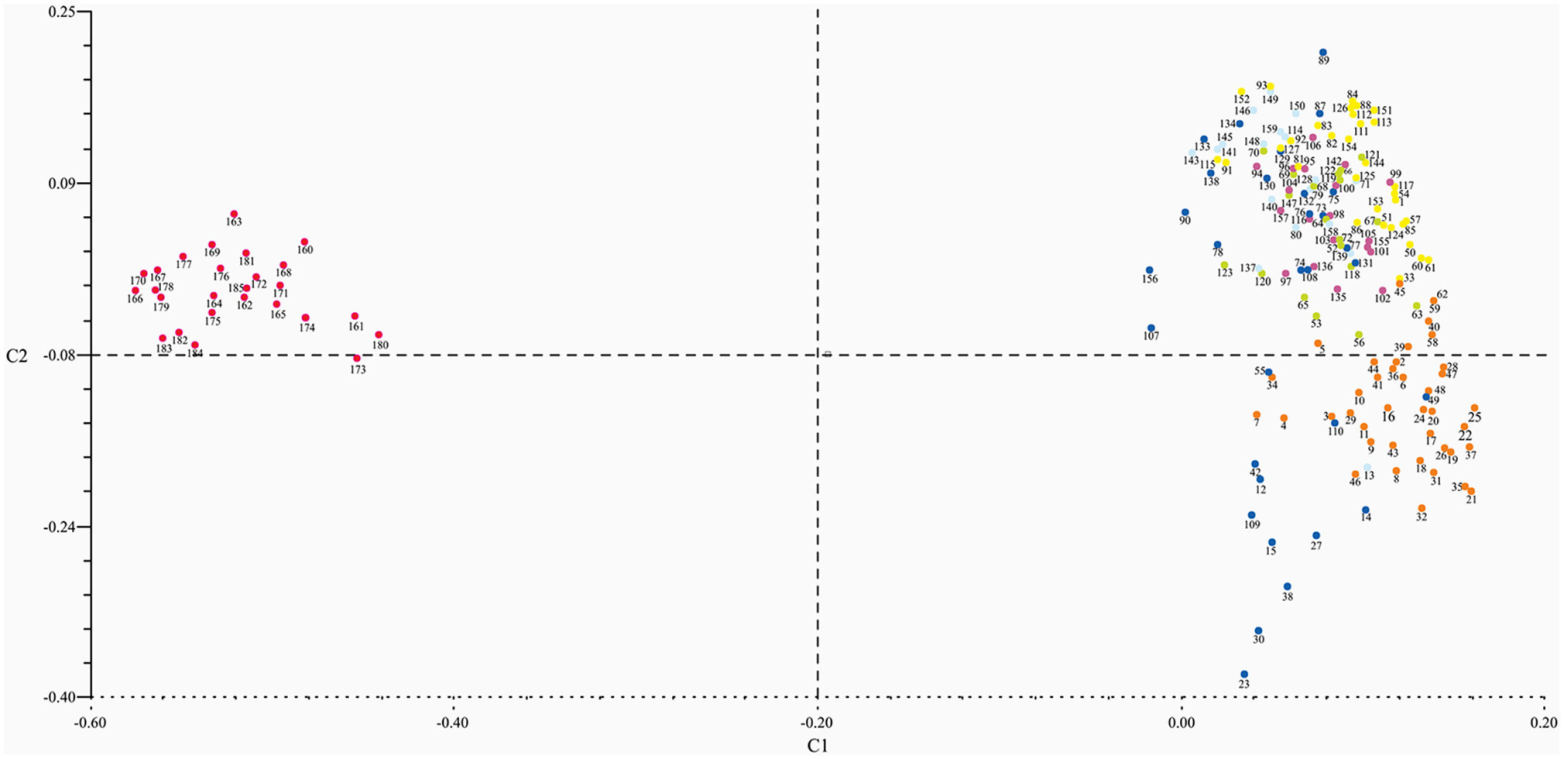
**Principal coordinate analysis showing the genetic relatedness of 185 cotton genotypes genotyped with SSR markers**. All *G. arboreum* genotypes were grouped in the upper left portion and *G. hirsutum* genotypes in the upper and lower portion of the graph. Dots color corresponds to different clusters (red = cluster-I; dark blue = cluster-II; yellow, green, purple, and light blue = cluster-III and orange = cluster-IV).

Principle coordinate analysis (PCoA) grouped the genotypes into four clusters which were consistent with the number of clusters calculated using UPGMA analysis (through Power marker). Out of four clusters, three were of *G. hirsutum* genotypes. All the *G. arboreum* genotypes belonging to cluster-I (as deduced by UPGMA analysis through Power marker) were distributed in the upper left portion of the resulted graph (Figure 6).

The genotypes in Cluster-II were distributed in the upper and lower right portion of the graph. One *Bt*-cotton genotype “CEMB-2” was not in parsimony with the other members of this group. Unlike UPGMA clustering, PCoA scattered the genotypes. The cluster-III comprised of 89 genotypes which were distributed in the upper right portion of the resulted plot. These clusters were inconsistent with the pattern of clustering of genotypes done through UPGMA analysis. For example, “FC 4245”—grouped in cluster-III using UPGMA analysis while this genotype did not join the cluster-III using the PCoA. Cluster-IV, the second largest cluster, comprised of 39 genotypes which were distributed in the lower right portion of the plotted graph. This cluster followed the same pattern as in UPGMA. All clusters were tightly bound in comparison with cluster-II—illuminating the presence of diverse genotypes in this cluster.

### Structure analysis

**Figure 7:**
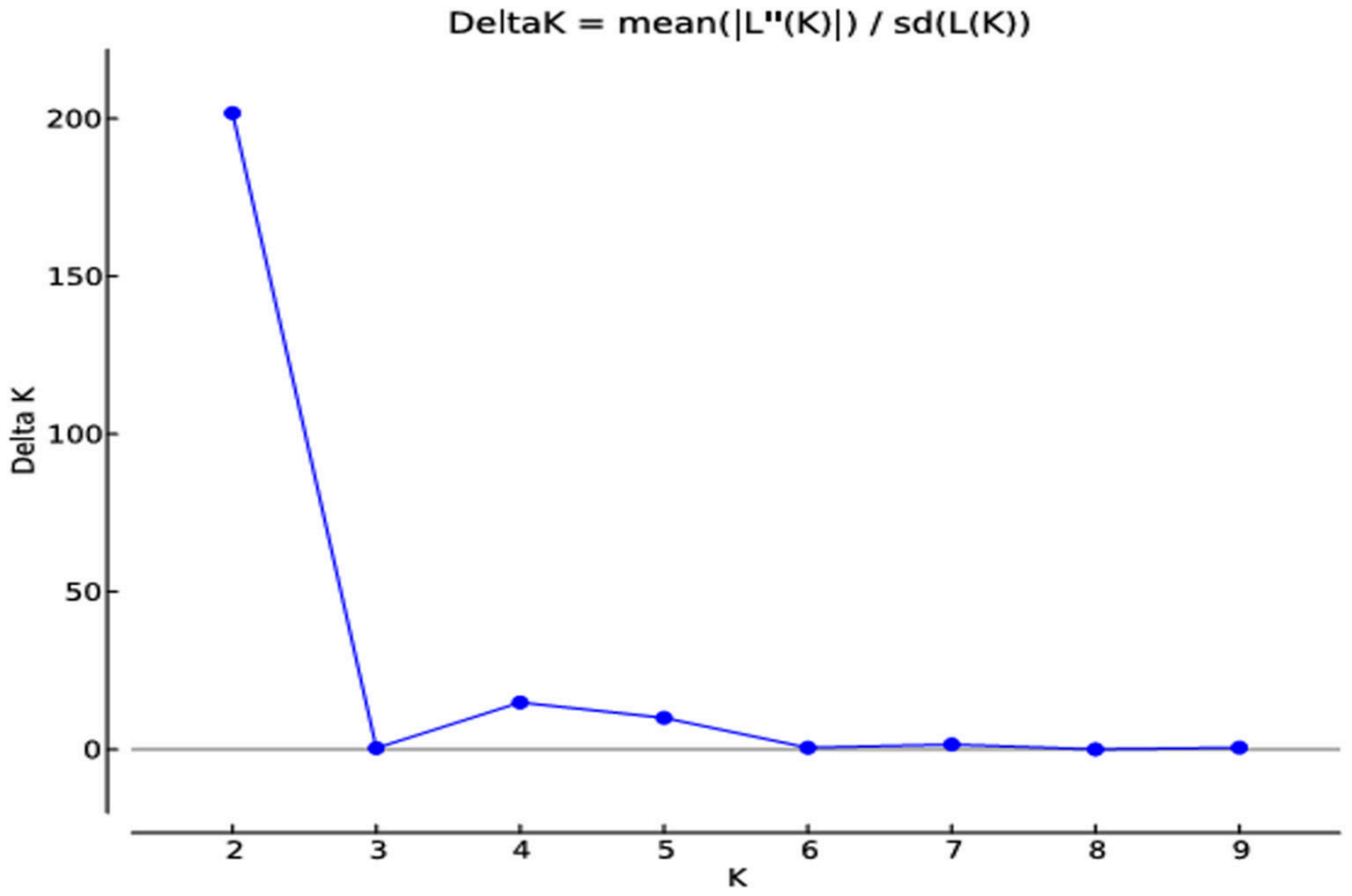
**Estimates of subpopulations using delta K-values, ranged K-values from 2 to 9 in 185 cotton genotypes by deploying a method proposed by Evanno et al. (2005)**.

The analysis based on genetic distances and also on the population structure revealed that cotton genotypes understudy had substantial population structure. Cotton genotypes were assigned to subpopulations based on maximum likelihood and ΔK-values. The maximum ΔK score (2 and 4) was calculated. Graphical presentation of K clusters against ΔK also strengthened the aforementioned results (Figure 7).

The results obtained through STRUCTURE software were in accordance with the results obtained through UPGMA analysis based on the genetic distances but the number of genotypes varied in each of the clusters obtained through UPGMA analysis. Mean value of alpha was found 0.0318 at *K* = 4. At *K* = 2, all 185 genotypes were grouped into two major clusters viz: *G*. *hirsutum* and *G. arboreum* through structure analysis. First cluster was comprised of 159 genotypes of *G. hirsutum* and the second cluster consisted of 26 genotypes of *G.arboreum*. No admixture was found for genotypes of both the species (Supplementary Figure 3).

**Figure 8:**
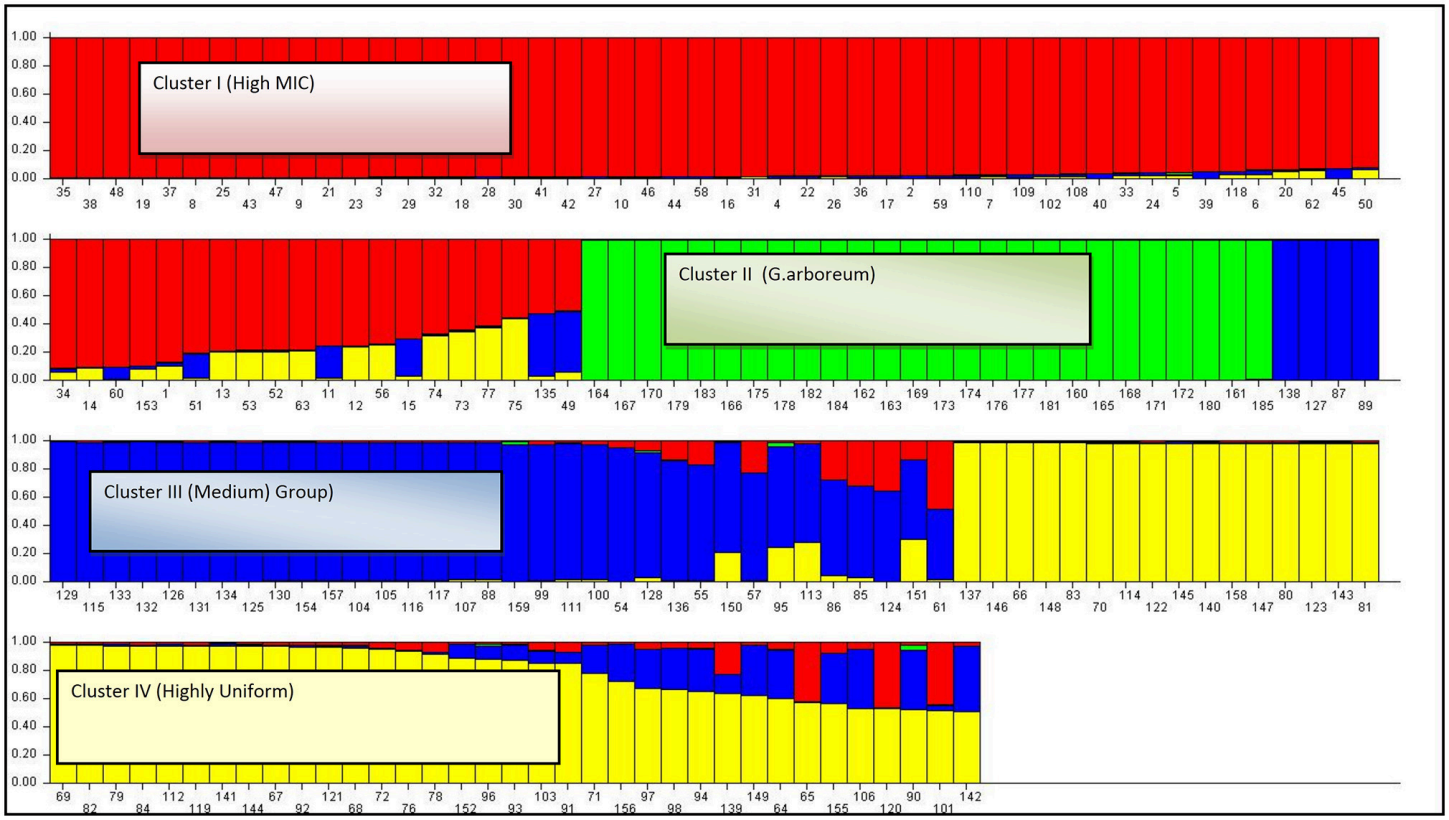
**The summary plot of Q-matrix estimates**. cluster 1—High MIC germplasm group (defined with the red color); cluster 2—*G. arboreum* germplasm group (defined with the green color); cluster 3—medium germplasm group (defined with the blue color); cluster 3—Highly uniform germplasm group (defined with the yellow color).

After increasing the K-value (*K* = 4), composition of *G. arboreum* cluster remained the same. While the genotypes of *G. hirsutum* cluster were further sub-grouped into three clusters, viz: cluster-I (genotypes with high Micronaire value), cluster-III (genotypes grouped in medium staple length), and cluster-IV (genotypes produced lint with high uniformity index). Model based approach revealed the ancestry information among groups (Figure 8).

In total 185 genotypes were assigned to four specific clusters with a minimum probability of 0.53. A total of 70 genotypes were assigned to cluster-I. Out of these, 54 genotypes were found to be homogeneous while 16 genotypes showed admixture with the other clusters. In total, four genotypes shared common ancestry with the genotypes grouped in cluster-III, eleven genotypes with cluster-IV, and only one genotype shared mixed ancestry with the cluster-III and IV. A total of 26 genotypes were assigned to cluster-II. All the genotypes of *G. arboreum* joined this cluster. No admixture was found within this cluster.

Cluster-III comprised of 38 genotypes. Out of these, 27 were homogeneous while 11 genotypes were found admixtures with the other clusters. In total, seven genotypes shared common ancestry with the genotypes of cluster-I, three genotypes with cluster-IV, and only one genotype from this cluster shared ancestry with cluster-I and IV. A total of 51 genotypes were assigned to cluster-IV. In total, 31 genotypes were found homogeneous while 20 genotypes showed admixture with the other clusters. A total of three genotypes shared common ancestry with the genotypes of cluster-I, eight with cluster-III while nine genotypes shared mixed ancestry with cluster-I and III.

### Linkage disequilibrium (LD)

**Figure 9:**
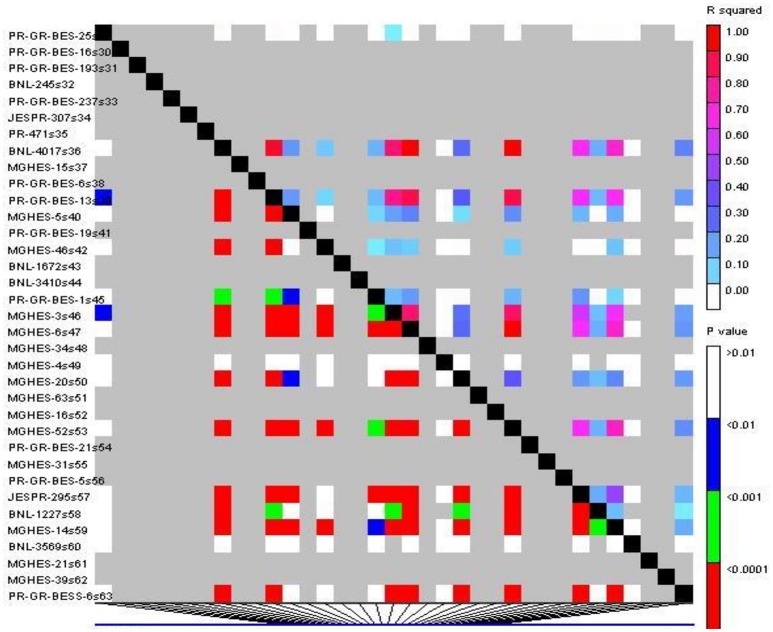
**The triangle LD plot for a pairwise genome-wide LD between SSR loci**. Polymorphic SSR primer pairs were plotted on both X-axis and Y-axis. Each pixel above the diagonal represents the *r*^2^ size of the corresponding pairs of markers, as shown in the color code at the upper right, and each pixel below the diagonal represents the *P*-value size of testing the LD at the lower right.

The linkage disequilibrium (LD) of the studied genotypes was determined using 95 pair of SSR markers. In total 6.8% (*r*^2^ ≥ 0.05) and 4.4% (*r*^2^ ≥ 0.1) of the marker pairs based on *r*^2^ estimates showed significant LD. LD was unevenly distributed on each chromosome and LD level concentrated on chromosome No. 9,14, 20, and 26. Triangle plots for pairwise LD between SSR markers demonstrated significant LD blocks in the genome-wide LD analysis (Figure 9).

### Association analysis between fiber traits and SSR markers

A total of 13 markers associated with the average boll weight were identified. Out of these, 12 were identified using GLM while only one was identified by exposing the data to MLM. The associated markers were identified on chromosome No. 7, 9, 13, 14, 15, 19, 20, 25, and 26. A very firm association for average boll weight was observed for MGHES-15 (*P* = 3.538E-07) which was mapped on chromosome No. 14. By deploying the MLM, only one marker PR-25, mapped on chromosome No. 24, showed the association with this trait. The *P*-value for this marker was 7.10E-04 and the *r*^2^-value was 0.065. A total of 18 associated markers with GOT percentage were identified using the GLM alone. These markers were identified on chromosome No. 7, 9, 13, 14, 15, 17, 19, 20, 21, 24, 25, and 26. Strong associations for the GOT percentage were observed with MGHES-53 and PR-21 having *P*-value (6.717E-47) which were mapped on chromosome No. 13 and 26, respectively.

In total, eight SSR markers were found to be associated with micronaire value using the GLM. Associated markers were located on chromosome No. 7, 13, 14, 15, 20, and 26. A very firm association of the micronaire value was observed with BNL-1227 having *P*-value of 1.81E-04 which was mapped on chromosome No. 26. A total of two common markers PR-25 and MGHES-67 associated with the staple lenght were identified using both the GLM and MLM at a standard *P* = 0.0001. A total of 18 significant marker trait associations were identified using GLM while only two were identified by exposing the data to MLM. The associated markers were present on chromosome No. 7, 9, 13, 14, 15, 17, 19, 20, 21, 24, 25, and 26. Strong associations were identified for MGHES-53 (mapped on chromosome No. 13) and PR-21 (mapped on chromosome No. 26), having *P*-value (3.13E-46). By deploying the MLM, only two associated markers, e.g. MGHES-67 with *P*-value of 2.55E-04 and *r*^2^ values of 0.110, located on chromosome No. 19, and PR-25 with *P*-value of 8.55E-04 and *r*^2^-value of 0.091, on chromosome No. 24 were identified. These two markers MGHES-67 and PR-25 showed marker trait association in both GLM and MLM models.

A common marker MGHES-51 (LOD = 2) associated with the fiber bundle strength was identified using GLM and MLM. The associated marker was present on chromosome No. 15. MGHES-51 showed association with fiber bundle strength with *P*-value of (3.57E-03) and *r*^2^ of (0.045) using the GLM while with MLM, the marker showed 9.19E-03 and 0.038, *P*-value, and *r*^2^, respectively. By exploiting the GLM, BNL-3408 was found associated with the fiber bundle strength having *P*-value (9.14E-03) and *r*^2^ (0.112), and it was located on chromosome No. 17. By deploying MLM, PR-25 (mapped on chromosome No. 24) showed association with fiber bundle strength having *P*-value (4.15E-03) and *r*^2^ (0.046).

Only one common marker (PR-25) associated with the UI was identified using GLM as well as MLM. However, a total of 15 markers associated with UI were observed by deploying the GLM model alone. A very high association was shown by MGHES-53 (mapped on chromosome No. 13) and PR-21 mapped on chromosome No. 26) having same *P*-value (1.03E-17). By deploying MLM, only a PR-25 showed association with the fiber uniformity (*P*-value, 1.35E-04) with *r*^2^-value of 0.121, located on chromosome No. 24. Further details of each of the marker's association with each of the traits are presented in Table 5. An overview of marker-trait associations for the studied traits is given in Table 6.

**Table 5:** **Association of Marker loci with agronomic and fiber quality traits and their chromosome number**.

**Traits**	**Marker name**	**Chr. no**.	**GLM(Q)**		**MLM(Q+K)**	
			***P*****-value**	***r*****^2^**	***P*****-value**	***r*****^2^**
Average boll weight	MGHES-36	7	0.000119	0.086	–	–
	MGHES-34	9	2.95E-05	0.099	–	–
	MGHES-53	13	1.05E-05	0.102	–	–
	MGHES-15	14	3.54E-07	0.148	–	–
	MGHES-5	14	0.000141	0.087	–	–
	MGHES-51	15	0.00018	0.075	–	–
	BNL-3569	19	9.76E-05	0.098	–	–
	MGHES-55	20	0.000403	0.067	–	–
	PR18	20	3.07E-05	0.109	–	–
	PR-25	24	–	–	7.10E-04	0.065
	PR-2	25	0.000405	0.067	–	–
	PR-21	26	1.05E-05	0.102	–	–
	BNL-1227	26	7.9E-06	0.111	–	–
GOT %	MGHES-36	7	7.52E-43	0.635	–	–
	MGHES-6	9	1.76E-24	0.341	–	–
	MGHES-34	9	2.81E-45	0.644	–	–
	MGHES-53	13	6.72E-47	0.636	–	–
	MGHES-15	14	1.33E-41	0.630	–	–
	MGHES-5	14	3.93E-36	0.585	–	–
	PR-19	14	3.62E-08	0.145	–	–
	MGHES-51	15	6.72E-28	0.454	–	–
	BNL-3408	17	1.33E-06	0.203	–	–
	BNL-3569	19	1.03E-31	0.511	–	–
	MGHES-67	19	2.34E-10	0.187	–	–
	MGHES-55	20	8.3E-27	0.440	–	–
	PR-18	20	1.25E-45	0.637	–	–
	MGHES-63	21	6.38E-11	0.197	–	–
	PR-25	24	2.96E-12	0.221	–	–
	PR-2	25	0.000109	0.075	–	–
	PR-21	26	6.72E-47	0.636	–	–
	BNL-1227	26	4.79E-44	0.624	–	–
Micronaire value	MGHES-36	7	5.08E-04	0.068	–	–
	MGHES-53	13	2.56E-04	0.068	–	–
	MGHES-15	14	8.47E-04	0.064	–	–
	MGHES-51	15	3.97E-04	0.064	–	–
	MGHES-55	20	5.80E-04	0.060	–	–
	PR-18	20	7.18E-04	0.073	–	–
	PR-21	26	2.56E-04	0.068	–	–
	BNL-1227	26	1.81E-04	0.075	–	–
Staple length	MGHES-36	7	8.99E-42	0.632	–	–
	MGHES-6	9	5.14E-27	0.380	–	–
	MGHES-34	9	6.84E-41	0.616	–	–
	MGHES-53	13	3.13E-46	0.630	–	–
	MGHES-15	14	7.63E-44	0.651	–	–
	MGHES-5	14	1.38E-37	0.600	–	–
	PR-19	14	1.41E-08	0.153	–	–
	MGHES-51	15	1.80E-32	0.505	–	–
	BNL-3408	17	3.87E-11	0.319	–	–
	BNL-3569	19	3.82E-31	0.504	–	–
	MGHES-67	19	2.03E-15	0.275	2.55E-04	0.110
	MGHES-55	20	1.42E-23	0.398	–	–
	PR-18	20	7.37E-45	0.630	–	–
	MGHES-63	21	5.39E-09	0.161	–	–
	PR-25	24	2.26E-06	0.109	8.55E-04	0.091
	PR-2	25	2.88E-08	0.147	–	–
	P-21	26	3.13E-46	0.630	–	–
	BNL-1227	26	1.80E-43	0.623	–	–
Fiber bundle strength	MGHES-51	15	3.57E-03	0.045	9.19E-03	0.038
	BNL-3408	17	9.14E-03	0.112	–	–
	PR-25	24	–	–	4.15E-03	0.046
Uniformity index	MGHES-36	7	1.69E-16	0.339	–	–
	MGHES-6	9	1.18E-10	0.208	–	–
	MGHES-34	9	9.63E-17	0.336	–	–
	MGHES-53	13	1.03E-17	0.332	–	–
	MGHES-15	14	1.77E-15	0.324	–	–
	MGHES-5	14	4.95E-15	0.317	–	–
	PR-19	14	0.000442	0.066	–	–
	MGHES-51	15	3.07E-10	0.196	–	–
	BNL-3569	19	3.87E-15	0.307	–	–
	MGHES-55	20	9.02E-11	0.207	–	–
	PR-18	20	1.17E-16	0.333	–	–
	MGHES-63	21	2.23E-07	0.137	–	–
	PR-25	24	5.15E-17	0.321	1.35E-04	0.121
	PR-21	26	1.03E-17	0.332	–	–
	BNL-1227	26	2.07E-16	0.323	–	–

**Table 6:** **Overview of marker–trait associations between different traits**.

**Sr. No**.	**Markers name**	**Average boll weight**	**GOT %**	**Micronaire value**	**Staple length**	**Fiber bundle strength**	**Uniformity index**
1	MGHES-5	√	√	×	√	×	√
2	MGHES-6	×	√	×	√	×	√
3	MGHES-15	√	√	√	√	×	√
4	MGHES-34	√	√	×	√	×	√
5	MGHES-36	√	√	√	√	×	√
6	MGHES-51	√	√	√	√	√	√
7	MGHES-53	√	√	√	√	×	√
8	MGHES-55	√	√	√	√	×	√
9	MGHES-63	×	√	×	√	×	√
10	MGHES-67	×	√	×	√	×	×
11	PR-2	√	√	×	√	×	×
12	PR-18	√	√	√	√	×	√
13	PR-19	×	√	×	√	×	√
14	PR-21	√	√	√	√	×	√
15	PR-25	√	√	×	√	√	√
16	BNL-1227	√	√	√	√	×	√
17	BNL-3408	×	√	×	√	√	×
18	BNL-3569	√	√	×	√	×	√

### Frequency of the favorable alleles

A total of 18 favorable alleles were detected. These alleles were divided into three classes. In the first class, the alleles with high frequencies were amplified by MGHES-53, MGHES-51, BNL-3569, MGHES-55, PR-25, PR-2, PR-21, PR-19, MGHES-67, and MGHES-63 in old as well as modern genotypes. In the second class, high frequencies of alleles amplified by MGHES-15, MGHES-5, PR-18, and MGHES-6 were detected in modern genotypes while with moderate frequencies in old genotypes. The third class of alleles (low to moderate frequencies) amplified by MGHES-36, BNL-1227, and BNL-3408 were observed in exotic as well as local *Bt*-cotton genotypes (Table 7).

**Table 7:** **Allele frequency for each favorable QTL Allele in historically released cotton genotypes**.

**Marker name**	**Exotic**	***Bt*****-cotton**	***G. arboreum***	**Cummulative**
MGHES-36	0.892	1.000	0.000	0.778
MGHES-34	0.942	0.850	0.923	0.940
MGHES-53	1.000	1.000	1.000	1.000
MGHES-15	0.885	0.950	1.000	0.908
MGHES-5	0.856	1.000	1.000	0.892
MGHES-51	1.000	1.000	1.000	1.000
BNL-3569	0.992	1.000	1.000	0.994
MGHES-55	1.000	1.000	1.000	1.000
PR-18	0.993	1.000	1.000	0.994
PR-25	1.000	1.000	0.750	0.940
PR-2	1.000	1.000	1.000	1.000
PR-21	1.000	1.000	1.000	1.000
BNl-1227	0.964	0.750	0.000	0.805
MGHES-6	0.842	1.000	1.000	0.881
PR-19	1.000	1.000	0.731	0.962
BNL-3408	0.561	0.050	0.000	0.427
MGHES-67	1.000	1.000	0.731	0.962
MGHES-63	0.957	1.000	0.423	0.886

## Discussion

Major breeding objective in cotton is to improve the yield and lint quality traits. The present breeding progress is slow than that of what was achieved in the past (1970 s). One of the major factors is the lack of diverse genetic resources for various traits of interest including quality traits (Rahman et al., 2008; Tyagi et al., 2014). In this regard, germplasm (tapped and untapped) available in Pakistan was collected from various cotton research institutes, and subjected to characterization for multiple traits. Thus a total of 546 exotic and locally bred cotton genotypes were selected for studying their response in 2009 at NIBGE Faisalabad. Out of these, 185 genotypes were selected on the basis of fiber characteristics (high and low quality). These genotypes included 139 exotic, 20 *Bt*-cotton and 26 *G. arboreum* (regionally adopted) genotypes which were sown for next 3 consecutive years (2011–2013) at three different locations NIBGE Faisalabad, CRS Vehari, and CCRI Multan.

### Screening of cotton genotypes for fiber quality traits

#### Mean performance of cotton genotypes

A number of traits including average boll weight, GOT percentage, micronaire value, staple length, fiber bundle strength, and uniformity index showed a wide range of phenotypic diversity among the cotton genotypes. In comparison between *G. hirsutum* and *G. arboreum* genotypes, all the studied traits were found superior in tetraploid genotypes as compared to diploid genotypes.

Mean value of average boll weight (2.88 g) was higher in exotic genotypes than that of *Bt*-cotton and *G. arboreum* genotypes. This study suggests that exploration of novel genes contributing to the average boll weight in exotic genotypes and then further incorporation of these genes in the cultivated genotypes will pave the way to increase average boll weight. The *Bt*-cotton genotypes exhibited the highest GOT percentage, staple length, fiber bundle strength, and uniformity index. The increase in the lint yield of *Bt*-cotton genotypes is due to the enhanced plant growth and number of bolls compared to the other genotypes (Dong et al., 2006), and or selection imposed for plants exhibiting high GOT percentage in the genetic material. Stable changing trend of each trait was exhibited over the years 2011–2013. The present investigation suggests that most breeding programs in Pakistan are focused on increasing the lint potential as well as lint quality traits (Rahman et al., 2002, 2005) for meeting the quality standards laid down by the Govt. of Pakistan.

#### Correlation studies

The correlated characters can be used for making indirect selection in cotton improvement. Like many other studies, negative correlation was observed for micronaire value with staple length and GOT percentage. These findings were supported by the previous researchers (Zhang and Guo, 2011). Positive correlation was observed for staple length with fiber bundle strength, uniformity index, average boll weight, and GOT percentage but the findings in one of the earlier study were different (Hussain et al., 2010). Average boll weight and GOT percentage were also found to be positively correlated in this study but in another study, negative correlation between average boll weight and GOT percentage was reported (Zhang Z. et al., 2011). Correlation among different traits suggest that there might be grouping distribution of QTLs for these traits (Zhang W. et al., 2011). Correlations among different traits may be helpful in selection to improve yield and fiber related traits all together. Major focus should be on the presence of heritable variability and favorable association among different traits to start any breeding program aimed at selection of favorable genotypes (Ali et al., 2008).

#### Principal component analysis

Multivariate analysis for studying a number of phenotypic triats in multiple crop species including cotton (Abbas et al., 2015; Shakeel et al., 2015), wheat (Janmohammadi et al., 2014), and maize (Mustafa et al., 2015) have been applied. In the present study, two clusters were developed using the first two principal components (El-Lawendey et al., 2008). Cultivated *Bt*-cotton genotypes, IR-NIBGE-3, IR-NIBGE-901, PGMB-3300, and AA-802 were found more divergent from rest of the *Bt*-cotton genotypes. In total, three genotypes LAT 27-588-1740, PRS-72, and Blanco-3363 (representing the exotic group) were diversed. These genotypes have unique morphological characters such as broad leaves (Personal communication).

#### Cluster analysis

Genotypes with similar traits were grouped in one cluster. For example, a total of 12 genotypes were clustered in cluster-I based on high fiber bundle strength (ranged from 29.28 to 30.20 g/tex)—relatively high than the general mean (27.67 g/tex). Mean value for micronaire, staple length and fiber bundle strength of four *Bt*-cotton genotypes (PGMB-3100, PGMB-1526, FH-113 and CEMB-2) was very close to mean of cluster-II—thus grouped in cluster-II. However, two *Bt*-cotton hybrids (GN-31 and GN-2085) were clustered with exotic genotypes as both were developed through crossing exotic cotton genotypes—a plausible explanation for grouping of these hybrids with the cluster of exotic cotton genotypes.

Mean value of each trait for *Bt*-cotton cluster was higher from the general mean, representing that these genotypes retain desirable agronomic traits, e.g., earliness, hairiness and better boll retention. For breeding *Bt*-cotton varieties in Pakistan, a diverse genetic resources containing *Bt-*gene was used as a donor parent followed by making three backcrosses or directly making selections in F_2_ generation (Zaman et al., 2015). Selection of diverse parent genotypes for making crosses offers opportunities to select for high yielding plants in F_2_ population (Suinaga et al., 2005). In the ancestory of few *Bt*-genotypes, both the high yielding genotypes (released as cultivars in other parts of the world) were used to breed for *Bt*-cotton genotypes in Pakistan—thus all favorable alleles for the desirable traits were pyramid in the resultant *Bt*-cotton genotypes—a convincing reason for escalating the means of traits than that of the general mean of the trial.

Cluster-IV contains all genotypes belonging to *G. arboreum*. Mean value of each of the six traits for genotypes of this cluster was less than that of general mean of each trait. It is obvious that the diploid A-genome genotypes (historically) produce the inferior lint fiber than that of the *G. hirsutum* genotypes. It is due to fiber-specific upregulation of *1-aminocyclopropane-1-carboxylic acid oxidase (ACO)* expression, as in the case of *G. hirsutum* and inactivation of ACO, as in *G. arboreum* might suppress fiber development (Li et al., 2014). Grouping of these genotypes in one cluster is because several reasons. First all genotypes belongs to the same species containing A-genome, thus all these genotypes exhibited similar traits. Secondly, these genotypes have been largely evolved in the drought prone region of Pakistan. These genotypes were bred largely by selection from the same gene pool. In earlier years, exchange of genetic material with the farmers/breeders was not common due to lack of large distance transportation means. Thus efforts were not made to introgress alleles from other cultivated cotton species (Rahman et al., 2007; Iqbal et al., 2015; Rahman, 2016).

### Genetic diversity

A number of markers including SSRs have been used extensively to study genetic diversity and also the development of genetic linkage maps in cotton (Mishra et al., 2013; Li et al., 2016a; Zhao et al., 2016). In this study, a total of 382 SSRs were used to survey the genome of 10 diverse genotypes of *G. hirsutum*. Selective genotyping reduces the cost by genotyping the most informative genotypes. A total of 95 SSR primer pairs out of 382 were found polymorphic, representing 25.74% polymorphism. Fluctuation in polymorphism percentage has been reported in earlier studies (Lin et al., 2005; Guo et al., 2007). The discrepancy in polymorphism percentage is due to different plant material, different number of SSRs/EST-SSRs from different tissues and number of primer pairs used (Tabbasam et al., 2014). For example, EST-SSRs (PIC = 0.010 for MGHES-19) found more conserved than that of SSRs (PIC = 0.726 for BNL-1672), with a mean value of 0.175. Fluctuation in PIC-values was reported in multiple studies (Abdurakhmonov et al., 2008)—originating largely from the type of marker used, number of genotypes surveyed, type of germplasm used and location of marker (Bardak and Bolek, 2012). Markers with high PIC-values offer greater opportunity for breeding cotton with excellent genetics (Bashir et al., 2015).

The selected SSRs amplified alleles ranged from 01 to 06 with a mean value of 1.71 alleles/marker. Significant variations in allele numbers per marker have been found in cotton (Liu et al., 2000; Bardak and Bolek, 2012), rice (Garris et al., 2005), and wheat (Oliveira et al., 2012)—such variations are originating from the type of markers used, germplasm to be genotyped and also the platform used for the resolution of the amplified products (Lacape et al., 2007).

Reports on calculation of genetic diversity among the cotton genotypes bred in Pakistan are scanty (Rahman et al., 2007; Dahab et al., 2013; Saeed F. et al., 2014). In the present study, a large number of representative cotton genotypes were characterized using SSR—calculated narrow genetic base, i.e., genetic dissimilarity coefficients ranged from 0.003 to 0.235 with a mean value of 0.098. “DC-116” (*G. arboreum*) was found the most genetically diverse (0.163). It is more likely that “DC-116” originated from a distinct population. Limited genetic diversity has also been observed in multiple reports (Rahman et al., 2007; Kalivas et al., 2011)—suggesting that bottleneck in evolution occurred in cotton germplasm (Iqbal et al., 2001). Fluctuation in genetic diversity estimates stems from type and number of marker surveyed and germplasm used. The other reason of low genetic diversity is due to excessive use of highly adaptive germplasm in breeding program. Thus efforts should be made to incorporate genes from wild sources into the cultivated varieties to enhance the genetic base of the newly developed varieties (Rahman et al., 2007).

More genetic variations among the exotic germplasm was depicted through UPGMA tree as compared to the locally cultivated varieties. Low genetic variation among the modern cotton genotypes is due to the extensive use of improved cotton genotypes in developing new varieties (Brown-Guedira et al., 2000). For example, breeders prefer to add new traits by crossing with the adapted donor parent. Second choice would be the use of germplasm of the corresponding species rather than using the wild sources or other distantly related parent genotypes for avoiding the introgression of negative characters from the unadapted parent genotypes (Rahman et al., 2002). Molecular diversity analysis using PCoA grouped the studied genotypes into four major clusters which are in line with results of UPGMA. Cluster-II comprising of exotic genotypes showed more variation as compared to the other clusters. These findings strengthened the results obtained through UPGMA.

#### Population structure

In the present studies, 47 (25.41% of the total) genotypes showed shared ancestry (alpha = 0.0318). Such commonalities have also been elucidated in multiple reports (Zeng et al., 2009; Tyagi et al., 2014). Sharing of cotton germplasm among the breeders or due to the frequent use of few lines having favorable agronomic traits are the most plausible reasons of shared ancestory (Van Esbroeck and Bowman, 1998). The phenomena of sharing of germplasm is more frequent within the region of adaptation than that of across the continents. However, later option seems appropriate to breed cotton genotypes with diverse genetic base.

The differentiation between subpopulations of the present studies was further confirmed by calculating high FST-value (0.24–0.73). However, substantial fluctuations in FST-values have been reported for different crop species including cotton (0.29–0.42) (Tyagi et al., 2014), rice (0.20–0.46) (Courtois et al., 2012), and corn (0.06–0.31) (Liu et al., 2003).

All the aforementioned approaches (UPGMA, PCoA, and STRUCTURE) for calculating population structure revealed almost positive correlation between population structure and geographic eco-types. In the present studies, most genotypes with common pedigree were clustered together. However, there were few discrepancies as some genotypes did not cluster according to the given pedigree. It is much likely that breeders claimed dubious/false pedigree of cotton varieties or sometime cross pollination or mixing at ginning machines may be another cause of disagreement with the claimed pedigree. Similar findings have been documented in previous reports where discrepancies were exhibited between pedigree information and SSR based genetic relationship (Fang et al., 2013).

### Linkage disequilibrium (LD)

Calculation of LD is a prerequisite before conducting association mapping studies. In this study 6.8% SSR markers pairs exhibited significant LD at *r*^2^ ≥ 0.05. Fluctuations in LD-value have been reported in multiple cop species including cotton (Abdurakhmonov et al., 2008, 2009; Saeed M. et al., 2014). Appearance of long haplotypic blocks in the present studies is due to selection pressure imposed by the breeders for desirable traits—resulted in accumulation of favorable genes in most cotton genotypes. Secondly, in Pakistan, cotton is dominantly a self-pollinated crop—consequently the recombination frequency will be reduced than that of cross pollinated crops.

### Association mapping

Association mapping is a promising tool that can be deployed for gene identification—responsible for complex traits. However, several factors affect the efficiency of association mapping including nature of the trait and lack of high quality molecular markers—lagged behind than the other crop species like corn. In the present studies, association mapping approach was used to find DNA markers associated with the fiber quality traits.

Various studies illuminated the importance of kinship because it produces LD between genetically linked as well as unlinked loci as it was demonstrated in maize (Stich et al., 2005). These results indicated that kinship matrix should be considered for association mapping in order to lessen the false positive associations. In this study, SSR markers were used to assess the marker trait associations because SSR/SNPs markers are preferred in GWAS over the other marker assays (Remington et al., 2001; Zhao et al., 2016). In most of our results, *P-*values were found lower in GLM than the MLM. Such findings were also demonstrated in another study (Yu et al., 2011).

In this study, 75 marker trait associations were identified. A number SSR markers (18) were associated with more than one trait. For example, MGHES-51 was found associated with multiple traits including average boll weight, GOT percentage, micronaire value, staple length, fiber bundle strength, and uniformity index. It is suggested that one locus may be involved in conferring multiple traits, which is the result of gene–gene interactions or pleiotropism (Lehner, 2011). Moreover, MGHES-51 is an EST-SSR. EST-SSRs (SSRs derived from coding sequences are called EST-SSRs (Saha et al., 2003) have more potential to identify changes in the genes accumulated during domestication (Wang et al., 2007). In another study, a total of 70 marker-trait associations in 99 *G. hirsutum* accessions were reported using 97 polymorphic SSR markers. Elite alleles for fiber related traits were explored in various germplasm accessions—increases the potential of SSR markers for utilizing in marker assisted breeding to improve fiber quality (Cai et al., 2014). Variation in population structure, QTL detection methods and environmental conditions restrict our choice to compare the newly identified QTLs with the already reported QTLs (Lakew et al., 2013).

A total of 13 new SSR markers were linked with average boll weight. In earlier studies, a total of three different SSR markers (NAU2272, NAU2439, and NAU3084) associated with average boll weight mapped on chromosomes No. 14, 24, and 26 were reported (Zhang et al., 2013). In another study, a total of 19 QTLs were reported on seven chromosomes using 178 recombinant inbred lines (Wang et al., 2015). A total of 18 SSR markers were associated with GOT percentage. These markers are different from the earlier reported markers (Zhang et al., 2013) but are mapped on same chromosomes (1, 2, 10, 14, 16, 17, 18, 19, 22, 23, 24, 25, and 26) at different positions. In Pakistan, breeders usually select advanced lines/varieties with lint depicting micronaire value in the range of 3.5–4.9 (Rahman et al., 2014). In this study, a total of eight marker-trait associations for micronaire value were found by deploying GLM approach only and association was not found using MLM approach. Chromosome No. 20 contains QTLs which determine micronaire value. In another study, QTLs conferring micronaire value were also identified on this chromosome (Zhang et al., 2013). Staple length has always been considered as a major contributor to yarn strength and processing performance. In the present study, a total of 18 SSR markers were associated with staple length which were mapped on chromosome No. 7, 9, 13, 14, 15, 17, 19, 20, 21, 24, 25, 26. In another study, a total of 17 marker-trait associations with staple length were reported on the same chromosomes (9, 13, 14, 19, 20, 24) but on different position (Cai et al., 2014). In total, three SSRs were found associated with the fiber bundle strength. Chromosome No. 24 was the common chromosome which was also reported in earlier association mapping studies (Cai et al., 2014). Fluctuation in chromosome number related to fiber bundle strength was also reported (Wang et al., 2006). The discrepancy in findings may arise from using different population i.e., *G. hirsutum* intraspecific biparental population. BNL-3408 associated with GOT percentage, staple length, and fiber bundle was the common marker which was also reported in previous association mapping studies (Zeng et al., 2009; Kalivas et al., 2011). This marker can be used confidently to identify genotypes with high lint potential and fiber quality traits across the different cotton growing countries. BNL-3408 can also be used as a specie specific primer and it was validated by another studies (Tabbasam et al., 2014). A total of 15 SSR markers were associated with the uniformity index. These markers were different but mapped on same chromosomes (7, 13, 14, 16, 25) at different positions (Sun et al., 2012). Fluctuation in chromosome number related to uniformity index was also reported in previous studies (Zhang et al., 2013). The difference in results may be due to the use of different types of population e.g., populations developed using 4-way cross, 3-way cross, or bi-parental population and or developed by crossing two different cotton species.

#### Frequency of favorable alleles

Most upland cotton genotypes have been bred from a limited gene pool—retarding the future breeding progress and increasing the potential risk of vulnerability to fluctuating climatic conditions. Breeders have substantially fixed favorable alleles in newly bred cultivars by imposing selection of plants exhibiting high yield potential. Thus the other corresponding alleles on the same locus were rejected during the process of domestication. The alleles present at low to moderate frequencies in old genotypes/landraces should be brought under cultivation for improving the yield potential and quality traits.

### Implications and conclusions

In the current competitive scenario, it has been realized that conscious efforts should be made to improve the lint quality as well as GOT percentage without compromising the yield. In this regard, efforts are being made but the complexity of the trait is the main obstacle in improvement in lint quality. This data can be utilized by the cotton breeder for seeking help in planning crosses with the aim to improve the lint quality and GOT percentage. In the present studies, we identified one of the lines of IR-NIBGE-3701 (line No. 12) which showed high GOT potential while keeping intact the high yielding potential. This line was immediately multiplied and submitted for testing in national yield trials. This line out yielded all candidate lines and a standard cotton variety CIM-496. Another future line, PGMB-3300 (identified in this study) which has very long staple length and its seed has been distributed to different national research organization for multiplication. The analysis of such large data has laid down the foundation of information relating the association of traits which are useful for varietal selection in breeding projects. At the genomic front, this data has been utilized for tagging QTLs conferring fiber traits. Multiple mapping approaches, such as QTL mapping and association mapping—evolved very rapidly over the last three decades, offer advantages for identifying the important QTLs conferring various traits of interest in multiple plant species. The present studies validated the associations of fewer makers drawn previously on bi-parental populations. Thus the aforementioned markers validated using the association mapping studies can be used in breeding for high quality traits. Since this study is novel, and thus can also be used in various national and international genomic projects for cloning of the important genes. The finding of the present study could pave the way to understand the genetic pathways underlying the fiber quality in cotton and could open new doors to find the putative regions of the genes controlling fiber quality by deploying different bioinformatics tools.

## Author contributions

MR conceived the study. MI performed the experiments and data analysis.

### Conflict of interest statement

The authors declare that the research was conducted in the absence of any commercial or financial relationships that could be construed as a potential conflict of interest.
