# Supplementary material for: Identification of Marker-Trait Associations for Lint Traits in Cotton
Source: Front Plant Sci. 2017 Feb 6;8:86. doi: 10.3389/fpls.2017.00086 (PMC5292784; doi:10.3389/fpls.2017.00086)
Supplement: Supplementary file 1 [file Table1.DOCX]

Supplementary Table 1: List of cotton genotypes with parentage/accession number

| Sr # | Genotypes name | Genome | Chr.# | Parentage/Accession # | |
| --- | --- | --- | --- | --- | --- |
| *Gossypium hirsutum* (Exotic) Genotypes | | | | | |
|  | G.S/LB-602 | AD | 52 | Exotic variety | |
|  | Lankart-57 | AD | 52 | Selection from Lankart | |
|  | LB-546 | AD | 52 | L/12 | |
|  | LAT 27-588-1740 | AD | 52 | L/40 | |
|  | LB-391 | AD | 52 | L/10 | |
|  | LA Okra 541 | AD | 52 | L/34 | |
|  | Lankart | AD | 52 | Indirect Sel. Petit Gulf | |
|  | Lambright GL-N | AD | 52 | Lambright GL-5/CA1786 | |
|  | Lakburn | AD | 52 | L/9 | |
|  | LA fregobract-2 | AD | 52 | L/37 | |
|  | G.hir.138F | AD | 52 | G/9 | |
|  | G 838 | AD | 52 | G/7 | |
|  | FC 4245 | AD | 52 | F/12 | |
|  | GAR F1-M1 | AD | 52 | G/28 | |
|  | F281 (g1) | AD | 52 | F/8 | |
|  | Empire Hc-Pl | AD | 52 | E/4 | |
|  | Early cot 31 | AD | 52 | Sel. CA491 | |
|  | Dung Ding | AD | 52 | D/55 | |
|  | E-302 | AD | 52 | E/7 | |
|  | Dunn 120 | AD | 52 | Sel. Tamcot SP23 | |
|  | Paymaster-909 | AD | 52 | PM 101/CA 2 | |
|  | PD 6520 | AD | 52 | P/25 | |
|  | Peking Cotton | AD | 52 | P/19 | |
|  | Pengze | AD | 52 | P/18 | |
|  | Philippine Cotton | AD | 52 | P/31 | |
|  | PRS-72 | AD | 52 | P/32 | |
|  | RA 31-9 | AD | 52 | R/51 | |
|  | Bayou SM- 1 | AD | 52 | Deltapine 15/Clevewilt 6 | |
|  | Bar 96/40 | AD | 52 | B/32 | |
|  | Belonga-5531 | AD | 52 | B/55 | |
|  | Blanco 3363 | AD | 52 | CA398/Lankart611 | |
|  | BjA-HL-27-B/163 | AD | 52 | B/58 | |
|  | Blight master A-5 | AD | 52 | Stormmaster//Stoneville 20/Acala 5675/3/Stormmaster | |
|  | Bambasa-49 | AD | 52 | B/46 | |
|  | Meads | AD | 52 | M/2 | |
|  | MCU-5 | AD | 52 | M/30 | |
|  | McNair 220 | AD | 52 | CKR 201/PD2165 | |
|  | Mex-I | AD | 52 | M/8 | |
|  | McNair 3150 | AD | 52 | McNair7125/CKR 310 | |
|  | Malawi A637xK4219 | AD | 52 | M/32 | |
|  | Locket-77 | AD | 52 | L/23 | |
|  | M11 | AD | 52 | Nectariless stock from J.R. Meyers | |
|  | Lumain No.1 | AD | 52 | ---- | |
|  | Delcot 277 NAF | AD | 52 | Rex//TJ/EF 310 | |
|  | Coker wild | AD | 52 | Delta type Weber/Lightning Express | |
|  | Copal-10 | AD | 52 | C/51 | |
|  | Culture 108-3 | AD | 52 | C/55 | |
|  | Dai 16 | AD | 52 | D/56 | |
|  | D-9 | AD | 52 | D/57 | |
|  | D-3-75 | AD | 52 | D/73 | |
|  | CA (68)36 | AD | 52 | C/11 | |
|  | BW-76-31DH | AD | 52 | B/54 | |
|  | B.T.K.12 | AD | 52 | B/7 | |
|  | Brace 67B | AD | 52 | B/56 | |
|  | BP-52 | AD | 52 | B/47 | |
|  | Brace 81/6 | AD | 52 | B/57 | |
|  | Bry cot-4 | AD | 52 | B/49 | |
|  | BPA-66 | AD | 52 | B/20 | |
|  | Carolina Queen (1) | AD | 52 | C/25 | |
|  | Cascot B-2 | AD | 52 | Sel. TX-Bonham | |
|  | Al reba/202 | AD | 52 | A/86 | |
|  | Albacala(69)32 | AD | 52 | A/61 | |
|  | ALK 4371/468 | AD | 52 | A/84 | |
|  | Albar A(57) 12 | AD | 52 | A/56 | |
|  | Allepo-40 | AD | 52 | Aleppo 1/ Acala SJ1 | |
|  | AL-SR 1054-2/302 | AD | 52 | A/87 | |
|  | Aldel-18 | AD | 52 | A/79 | |
|  | B-799 | AD | 52 | B/12 | |
|  | Arkugo c4 | AD | 52 | A/110 | |
|  | Babdal | AD | 52 | B/1 | |
|  | Arizona-6218 | AD | 52 | A/22 | |
|  | A.T.H.783 | AD | 52 | A/104 | |
|  | Austin | AD | 52 | A/1 | |
|  | ASA(65)38 | AD | 52 | A/45 | |
|  | AMSI-74 | AD | 52 | A/51 | |
|  | ICA-17NAF | AD | 52 | I/10 | |
|  | IM-216 | AD | 52 | I/11 | |
|  | Inta-SP-Toba-II | AD | 52 | I/5 | |
|  | Imperial Acala | AD | 52 | I/1 | |
|  | Kapal (A2106) | AD | 52 | K/2 | |
|  | Karnak 55 | AD | 52 | K/4 | |
|  | KapIana | AD | 52 | K/1 | |
|  | Kuanung | AD | 52 | K/3 | |
|  | L-142-9 | AD | 52 | L/16 | |
|  | Mex-13 | AD | 52 | M/28 | |
|  | MG-H-NE-523 | AD | 52 | M/50 | |
|  | MK-73 | AD | 52 | M/37 | |
|  | Misdale | AD | 52 | M/1 | |
|  | Muka (A641) | AD | 52 | M/35 | |
|  | Mex-8 | AD | 52 | M/12 | |
|  | Acala 1517-77 | AD | 52 | Acala 1517-70/unknown storm resistant | |
|  | AL-4-42/22 | AD | 52 | A/80 | |
|  | Afghanistan-1 | AD | 52 | A/109 | |
|  | AET-5 | AD | 52 | A/118 | |
|  | Accopaymaster | AD | 52 | A/35 | |
|  | AH( 67)M | AD | 52 | A/23 | |
|  | IAC- 12 | AD | 52 | I/7 | |
|  | Hybe 201 | AD | 52 | H/3 | |
|  | HL-1 | AD | 52 | H/4 | |
|  | Hopiacala | AD | 52 | Sel. no. 4447 from AHA 6-1-5 | |
|  | High gossypol germplasm mixture | AD | 52 | H/9 | |
|  | H-496 | AD | 52 | H/43 | |
|  | HG-18-45-N | AD | 52 | H/50 | |
|  | G.Var.Coker | AD | 52 | G/17 | |
|  | Gumbo Okra | AD | 52 | G/4 | |
|  | GSA-71 | AD | 52 | G/3 | |
|  | Greeg 25V | AD | 52 | G/1 | |
|  | Goa-1 | AD | 52 | G/34 | |
|  | Gossypol free seed | AD | 52 | G/2 | |
|  | GH-11-9-75 | AD | 52 | G/32 | |
|  | Genetic Cotton | AD | 52 | G/21 | |
|  | G.hir.S 2833 | AD | 52 | G/11 | |
|  | Paymaster-111 | AD | 52 | PM 101/Lankart 611 | |
|  | OK-86 | AD | 52 | O/1 | |
|  | PAN F3 5575 | AD | 52 | P/33 | |
|  | Northern. Star 4- 11 | AD | 52 | N/3 | |
|  | Mysora American | AD | 52 | M/29 | |
|  | Nectariless 73/33 | AD | 52 | N/11 | |
|  | NCS-S-1 x P/1-1 | AD | 52 | N/5 | |
|  | Mutant 189/75 | AD | 52 | M/38 | |
|  | N-69 | AD | 52 | N/9 | |
|  | HG-1 | AD | 52 | H/39 | |
|  | HAR 444-2-70 | AD | 52 | H/35 | |
|  | Huma-15 | AD | 52 | H/30 | |
|  | HA-7 | AD | 52 | H/16 | |
|  | H-SU-chow | AD | 52 | H/13 | |
|  | H 2918-2 | AD | 52 | H/8 | |
|  | Dos-056 | AD | 52 | D/64 | |
|  | DPL-16 | AD | 52 | D/24 | |
|  | Delcot2775 | AD | 52 | D/62 | |
|  | Delfos 6-343-6 | AD | 52 | D/2 | |
|  | DPL-SR-5 | AD | 52 | D/51 | |
|  | DT-Webber | AD | 52 | D/21 | |
|  | Coker 310(Fg) | AD | 52 | CKR 100 Staple/DP 15 | |
|  | Chung Mian | AD | 52 | C/49 | |
|  | Co2-P1 | AD | 52 | C/37 | |
|  | Cedix | AD | 52 | C/94 | |
|  | Acala 1517 E | AD | 52 | Acala3080/PD2165 | |
|  | A33-57 | AD | 52 | A/47 | |
| Gossypium hirsutum (*Bt*-cotton) genotypes | | | | |  |
|  | PGMB-3300 | AD | 52 | Selection from CIM-496 | |
|  | PGMB-1779 | AD | 52 | Selection from IR-CIM-443-14 | |
|  | PGMB-1408 | AD | 52 | VFK99/Exotic *Bt* source | |
|  | PGMB-3100 | AD | 52 | Selection from IR-NIAB-111 | |
|  | IR-NIBGE-3701 | AD | 52 | Selection from IR-CIM-448 | |
|  | IR-NIBGE-3 | AD | 52 | Selection from PGMB-2381 | |
|  | IR-NIBGE-901 | AD | 52 | FH 901/Bt source | |
|  | PGMB-1523 | AD | 52 | Selection from IR-NIBGE-2 | |
|  | PGMB-1526 | AD | 52 | Selection from IR-NIBGE-2 | |
|  | GN-31 | AD | 52 | Confidential | |
|  | GN-2085 | AD | 52 | Confidential | |
|  | AA-802 | AD | 52 | FH-1000 X HK-303 X LRA-5166 X LINEA-100 | |
|  | CEMB-1 | AD | 52 | ---- | |
|  | IR-NIBGE-1524 | AD | 52 | Selection from IR-NIBGE-2 | |
|  | AA-703 | AD | 52 | CIM-482 X HK-303 | |
|  | FH-113 | AD | 52 | CIM-448 X Bollgard® | |
|  | CEMB-2 | AD | 52 | ---- | |
|  | Sitara-008 | AD | 52 | Confidential | |
|  | MG-6 | AD | 52 | CIM-443 X IR-448 | |
|  | Neelum-121 | AD | 52 | A9-17 X EXOTIC-1 | |
| *G.arboreum* Genotypes | | | | |  |
|  | Rohi | A2 | 26 | Haroonabad local x D9 | |
|  | LDL-113 | A2 | 26 | Farmer field selection | |
|  | No.18 | A2 | 26 | Farmer field selection | |
|  | FDH-113 | A2 | 26 | Farmer field selection | |
|  | No-36 | A2 | 26 | Farmer field selection | |
|  | No-29 | A2 | 26 | Farmer field selection | |
|  | 212 | A2 | 26 | Farmer field selection | |
|  | Barnecum | A2 | 26 | Farmer field selection | |
|  | FDH-786 | A2 | 26 | Farmer field selection | |
|  | 451-R | A2 | 26 | Farmer field selection | |
|  | 23718 | A2 | 26 | Farmer field selection | |
|  | M-11 | A2 | 26 | Farmer field selection | |
|  | Garohill | A2 | 26 | Farmer field selection | |
|  | D-9 | A2 | 26 | Bahawalpur Desi-Selection | |
|  | HK-244 | A2 | 26 | Selected from local sector | |
|  | M-17 | A2 | 26 | Selected from local sector | |
|  | Synthetic | A2 | 26 | Selected from local sector | |
|  | FDH-228 | A2 | 26 | TD-1 x (comilla x FDH-170); 2002 | |
|  | No-17 | A2 | 26 | Selected from local sector | |
|  | Haroonabad | A2 | 26 | Farmer Selection | |
|  | 27518 | A2 | 26 | Selected from local sector | |
|  | 786 | A2 | 26 | Selected from local sector | |
|  | 231-R | A2 | 26 | Selection from 39 Mollisoni made in 1942 | |
|  | 450-R | A2 | 26 | Selected from local sector | |
|  | DC-116 | A2 | 26 | Selected from local sector | |
|  | No-35 | A2 | 26 | Selected from local sector | |

Supplementary Table 2: Mean performance of 185 cotton genotypes for average boll weight and ginning out turn (GOT) percentage.

| Sr # | Genotypes | Average boll weight (g) | | | | GOT percentage | | | |
| --- | --- | --- | --- | --- | --- | --- | --- | --- | --- |
|  |  | FSD | VH | MLN | Ave. | FSD | VH | MLN | Ave. |
| 1 | G.S/LB-602 | 3.04 | 2.99 | 3.12 | 3.05 | 38.15 | 36.54 | 37.54 | 37.41 |
| 2 | Lankart-57 | 2.79 | 3.05 | 3.39 | 3.08 | 37.99 | 36.94 | 36.12 | 37.02 |
| 3 | LB-546 | 2.56 | 2.78 | 2.63 | 2.66 | 35.62 | 33.3 | 34.89 | 34.6 |
| 4 | LAT 27-588-1740 | 2.32 | 2.61 | 3.05 | 2.66 | 30.2 | 28.78 | 31.53 | 30.17 |
| 5 | LB-391 | 3.03 | 2.72 | 3.25 | 3 | 40.23 | 38.19 | 36.54 | 38.32 |
| 6 | LA Okra 541 | 3.58 | 3.17 | 3.22 | 3.32 | 33.54 | 31.91 | 36.88 | 34.11 |
| 7 | Lankart | 2.41 | 2.21 | 2.46 | 2.36 | 37.93 | 35.44 | 34.19 | 35.85 |
| 8 | Lambright GLN(g1) | 3.39 | 2.71 | 3.17 | 3.09 | 38.6 | 34.11 | 35.85 | 36.19 |
| 9 | Lakburn | 3.31 | 2.89 | 3.06 | 3.09 | 34.07 | 32.92 | 35.88 | 34.29 |
| 10 | LA fregobract-2 | 1.90 | 2.58 | 2.95 | 2.48 | 37.36 | 36.49 | 36.08 | 36.64 |
| 11 | G.hir.138F | 2.86 | 2.73 | 2.82 | 2.8 | 38.37 | 37.06 | 37.33 | 37.59 |
| 12 | G 838 | 3.54 | 3.45 | 3.32 | 3.44 | 31.47 | 34.39 | 33.35 | 33.07 |
| 13 | FC 4245 | 2.91 | 2.24 | 2.81 | 2.65 | 37.84 | 34.55 | 31.69 | 34.69 |
| 14 | GAR F1 –M1 | 3.04 | 2.73 | 3.2 | 2.99 | 35.44 | 32.52 | 33.46 | 33.8 |
| 15 | F281 (g1) | 3.22 | 2.27 | 2.73 | 2.74 | 32.45 | 32.16 | 33.39 | 32.67 |
| 16 | Empire Hc-Pl | 3.09 | 2.79 | 3.36 | 3.08 | 42.35 | 41.57 | 38.15 | 40.69 |
| 17 | Early cot 31 | 3.79 | 2.56 | 3.52 | 3.29 | 36.33 | 34.29 | 34.96 | 35.2 |
| 18 | Dung Ding | 2.48 | 2.12 | 2.42 | 2.34 | 35.1 | 34.72 | 33.44 | 34.42 |
| 19 | E-302 | 3.79 | 3.35 | 3.35 | 3.49 | 40.16 | 37.48 | 36.49 | 38.04 |
| 20 | Dunn 120 | 2.39 | 2.93 | 3.3 | 2.87 | 37.61 | 38.4 | 37.82 | 37.94 |
| 21 | Paymaster-909 | 3.04 | 2.53 | 2.94 | 2.84 | 33.4 | 31.15 | 33.89 | 32.81 |
| 22 | PD 6520 | 2.55 | 2.19 | 2.61 | 2.45 | 39.94 | 38.14 | 35.45 | 37.85 |
| 23 | Peking Cotton | 3.07 | 3.36 | 3.3 | 3.24 | 32.4 | 30.84 | 33.3 | 32.18 |
| 24 | Pengze | 2.07 | 2.75 | 2.92 | 2.58 | 37.2 | 36.07 | 34.65 | 35.97 |
| 25 | Philippine Cotton | 3.21 | 2.95 | 2.93 | 3.03 | 38.94 | 35.64 | 34.64 | 36.41 |
| 26 | PRS – 72 | 2.69 | 2.69 | 3.35 | 2.91 | 33.78 | 34.08 | 34.32 | 34.06 |
| 27 | RA 31-9 | 2.97 | 2.6 | 2.82 | 2.8 | 36.53 | 33.54 | 34.06 | 34.71 |
| 28 | Bayou SM- 1 | 3.28 | 2.77 | 2.79 | 2.95 | 41.19 | 37.12 | 38 | 38.77 |
| 29 | Bar 96/40 | 3.06 | 3.13 | 3.14 | 3.11 | 36.82 | 35.18 | 34.78 | 35.59 |
| 30 | Belonga-5531 | 2.42 | 2.89 | 3.2 | 2.84 | 35.9 | 35.81 | 35.31 | 35.67 |
| 31 | Blanco 3363 | 3.51 | 2.57 | 3.14 | 3.07 | 27.68 | 29.57 | 32.83 | 30.02 |
| 32 | BjA-HL-27-B/163 | 3.10 | 2.82 | 2.93 | 2.95 | 32.22 | 33.06 | 32.36 | 32.55 |
| 33 | Blight master A-5 | 3.41 | 2.82 | 3.2 | 3.14 | 33.75 | 31.87 | 36.21 | 33.94 |
| 34 | Bambasa-49 | 3.16 | 2.88 | 2.87 | 2.97 | 33.94 | 31.8 | 33.75 | 33.16 |
| 35 | Meads | 1.71 | 2.07 | 2.75 | 2.18 | 30.76 | 30 | 32.77 | 31.18 |
| 36 | MCU-5 | 3.29 | 2.78 | 3.09 | 3.05 | 38.58 | 37.63 | 37.81 | 38.00 |
| 37 | MC-nair-220 | 3.19 | 2.36 | 2.98 | 2.85 | 36.82 | 35.73 | 37.23 | 36.59 |
| 38 | Mex-1 | 3.41 | 2.13 | 2.56 | 2.7 | 35.06 | 32.03 | 33.47 | 33.52 |
| 39 | MC-nair-3150 | 2.21 | 1.87 | 2.77 | 2.29 | 34.82 | 34.75 | 37.35 | 35.64 |
| 40 | Malawi A637xK4219 | 3.35 | 2.58 | 2.56 | 2.83 | 36.2 | 34.14 | 33.3 | 34.55 |
| 41 | Locket-77 | 2.62 | 1.68 | 2.49 | 2.26 | 30.71 | 28.99 | 33.75 | 31.15 |
| 42 | M- 11 (G. hir) | 3.07 | 2.84 | 2.77 | 2.89 | 37.96 | 32.91 | 33.92 | 34.93 |
| 43 | Lumain No.1 | 2.34 | 1.78 | 2.42 | 2.18 | 36.66 | 33.31 | 29.36 | 33.11 |
| 44 | Delcot 277 NAF | 2.77 | 2.75 | 2.91 | 2.81 | 35.8 | 34.78 | 33.4 | 34.66 |
| 45 | Coker wild | 1.58 | 1.51 | 1.84 | 1.64 | 34.1 | 32.58 | 33.52 | 33.4 |
| 46 | Copal-10 | 2.99 | 2.88 | 3.11 | 2.99 | 36.95 | 36.57 | 37.83 | 37.12 |
| 47 | Culture 108-3 | 3.61 | 3.29 | 3.43 | 3.44 | 33.44 | 31.6 | 31.33 | 32.12 |
| 48 | Dai 16 | 3.27 | 2.95 | 3.09 | 3.1 | 33.25 | 33.03 | 33.67 | 33.32 |
| 49 | D-9 (G.hir) | 3.23 | 2.77 | 2.93 | 2.98 | 37.6 | 36.38 | 36.86 | 36.94 |
| 50 | D-3-75 | 2.16 | 2.25 | 3.01 | 2.47 | 31.52 | 33.26 | 34.7 | 33.16 |
| 51 | CA (68)36 | 3.08 | 2.36 | 2.87 | 2.77 | 38.89 | 35.88 | 32.72 | 35.83 |
| 52 | BW-76-31DH | 3.07 | 2.5 | 2.65 | 2.74 | 34.81 | 33.88 | 36.9 | 35.2 |
| 53 | B.T.K.12 | 2.54 | 2.6 | 3.43 | 2.86 | 30.13 | 32 | 32.81 | 31.65 |
| 54 | Brace 67B | 3.65 | 2.97 | 3.51 | 3.38 | 37.77 | 37.24 | 37.11 | 37.37 |
| 55 | BP-52 | 3.11 | 2.52 | 2.62 | 2.75 | 35.77 | 32.81 | 32.11 | 33.56 |
| 56 | Brace 81/6 | 2.18 | 2.27 | 2.52 | 2.32 | 34.07 | 32.04 | 34.66 | 33.59 |
| 57 | Bry cot-4 | 3.3 | 2.57 | 2.96 | 2.94 | 35.35 | 32.29 | 35.96 | 34.53 |
| 58 | BPA-66 | 1.8 | 2.09 | 2.29 | 2.06 | 36.24 | 33.34 | 34.87 | 34.81 |
| 59 | Carolina Queen (1) | 2.44 | 2.07 | 2.57 | 2.36 | 34.67 | 34.03 | 35.11 | 34.6 |
| 60 | Cascot B-2 | 2.29 | 2.41 | 2.33 | 2.34 | 31.57 | 31.24 | 37.98 | 33.6 |
| 61 | Al reba/202 | 3.27 | 2.89 | 3.17 | 3.11 | 35.45 | 33.69 | 34.49 | 34.54 |
| 62 | Albacala(69)32 | 3.56 | 2.07 | 2.68 | 2.77 | 34.02 | 32.03 | 35.07 | 33.71 |
| 63 | ALK 4371/468 | 3.63 | 2.63 | 2.82 | 3.02 | 36.3 | 35.7 | 34.74 | 35.58 |
| 64 | Albar A(57) 12 | 1.9 | 2.34 | 2.67 | 2.3 | 37.46 | 35.92 | 36.72 | 36.7 |
| 65 | Allepo-40 | 3.27 | 2.56 | 3.1 | 2.98 | 34.95 | 34.38 | 37.28 | 35.54 |
| 66 | AL-SR 1054-2/302 | 3.44 | 2.55 | 3.17 | 3.05 | 35.64 | 33.59 | 33.01 | 34.08 |
| 67 | Aldel-18 | 2.5 | 2.48 | 2.78 | 2.59 | 34.55 | 33.13 | 34.01 | 33.9 |
| 68 | B-799 | 3.05 | 2.73 | 2.96 | 2.92 | 35.13 | 33.91 | 36.13 | 35.06 |
| 69 | Arkugo c4 | 2.87 | 2.19 | 2.43 | 2.49 | 37.57 | 36.15 | 34.12 | 35.95 |
| 70 | Babdal | 3.37 | 3.4 | 3.52 | 3.43 | 36.73 | 35.18 | 34.65 | 35.52 |
| 71 | Arizona-6218 | 1.63 | 1.76 | 2.36 | 1.92 | 34.66 | 32.09 | 36.2 | 34.32 |
| 72 | A.T.H.783 | 2.87 | 2.31 | 2.72 | 2.63 | 37.39 | 34.48 | 35.42 | 35.76 |
| 73 | Austin | 3.47 | 2.61 | 3.12 | 3.07 | 24.63 | 30.15 | 32.92 | 29.23 |
| 74 | ASA(65)38 | 3.39 | 2.51 | 2.78 | 2.89 | 40.67 | 39.24 | 34.93 | 38.28 |
| 75 | AMSI-74 | 2.99 | 2.49 | 2.78 | 2.75 | 34.83 | 32.47 | 35.82 | 34.37 |
| 76 | ICA- 17NAF | 3.82 | 3.14 | 3.54 | 3.5 | 33.87 | 33.18 | 32.64 | 33.23 |
| 77 | IM-216 | 3.3 | 2.22 | 2.76 | 2.76 | 35.87 | 36.04 | 34.79 | 35.57 |
| 78 | Inta-SP-Toba-II | 2.66 | 2.29 | 2.85 | 2.6 | 36.73 | 33.46 | 34.74 | 34.98 |
| 79 | Imperial Acala | 3.3 | 2.83 | 3.18 | 3.11 | 36.86 | 35.51 | 36.9 | 36.42 |
| 80 | Kapal (A2106) | 3.23 | 2.66 | 2.93 | 2.94 | 36.09 | 35.39 | 35.33 | 35.6 |
| 81 | Karnak 55 | 2.23 | 2.21 | 2.94 | 2.46 | 33.02 | 33.51 | 39.18 | 35.24 |
| 82 | KapIana | 2.77 | 2.47 | 2.86 | 2.7 | 35.77 | 34.83 | 35.82 | 35.47 |
| 83 | Kuanung | 3.41 | 3.17 | 2.99 | 3.19 | 33.7 | 33.54 | 34.2 | 33.81 |
| 84 | L1-42-9 | 3.12 | 2.5 | 2.81 | 2.81 | 37.82 | 35.97 | 35.88 | 36.56 |
| 85 | Mex-13 | 1.99 | 2.41 | 2.95 | 2.45 | 36.4 | 35.32 | 35.52 | 35.75 |
| 86 | MG-H-NE-523 | 3.82 | 3.18 | 3.4 | 3.47 | 34.87 | 35.79 | 35.51 | 35.39 |
| 87 | MK-73 | 3.25 | 2.61 | 3.15 | 3 | 38.9 | 35.23 | 35.26 | 36.46 |
| 88 | Misdale | 3.26 | 2.77 | 3 | 3.01 | 38.69 | 37.02 | 36.89 | 37.53 |
| 89 | Muka (A641) | 2.56 | 2.19 | 2.59 | 2.45 | 39.7 | 37.12 | 33.72 | 36.85 |
| 90 | Mex-8 | 3.58 | 2.88 | 3.42 | 3.3 | 33.01 | 32.79 | 35.45 | 33.75 |
| 91 | Acala 1517-77 | 3.82 | 3.56 | 3.48 | 3.62 | 34.94 | 34.61 | 35.92 | 35.16 |
| 92 | AL-4-42/22 | 2.13 | 2.73 | 3.04 | 2.63 | 32.54 | 31.59 | 34.8 | 32.98 |
| 93 | Afghanistan-1 | 3.57 | 2.11 | 3.03 | 2.91 | 41.34 | 36.92 | 34.06 | 37.44 |
| 94 | AET – 5 | 2.19 | 2.07 | 2.36 | 2.2 | 39.84 | 38.35 | 37.57 | 38.59 |
| 95 | Accopaymaster | 2.32 | 1.86 | 2.34 | 2.17 | 34.95 | 35.81 | 35.53 | 35.43 |
| 96 | AH( 67)M | 2.85 | 2.64 | 3 | 2.83 | 35.1 | 32.52 | 34.57 | 34.06 |
| 97 | IAC- 12 | 3.48 | 3.01 | 3.23 | 3.24 | 37.83 | 37.46 | 36.29 | 37.19 |
| 98 | Hybe 201 | 2.95 | 2.96 | 2.87 | 2.93 | 37.65 | 34.44 | 35.69 | 35.93 |
| 99 | HL-1 | 3.67 | 3.04 | 3.25 | 3.32 | 38.78 | 37.88 | 38.27 | 38.31 |
| 100 | Hopiacala | 2.61 | 2.35 | 2.79 | 2.58 | 34.76 | 30.95 | 32.08 | 32.6 |
| 101 | High gossypol germplasm | 3.22 | 2.52 | 2.83 | 2.86 | 40.23 | 38.49 | 34.97 | 37.9 |
| 102 | H-496 | 3.28 | 3.26 | 3.28 | 3.27 | 36.71 | 35.01 | 34.18 | 35.3 |
| 103 | HG-18-45-N | 3.36 | 3.01 | 2.89 | 3.09 | 35.84 | 33.98 | 34.7 | 34.84 |
| 104 | G.Var.Coker | 2.47 | 1.92 | 2.25 | 2.21 | 34.34 | 34.08 | 34.16 | 34.2 |
| 105 | Gumbo Okra | 2.86 | 2.62 | 2.99 | 2.82 | 34.98 | 32.55 | 34.63 | 34.05 |
| 106 | GSA-71 | 3.74 | 3.48 | 3.77 | 3.66 | 34.7 | 36.15 | 37.75 | 36.2 |
| 107 | Greeg 25V | 3.9 | 2.98 | 3.52 | 3.47 | 30.64 | 31.52 | 33.33 | 31.83 |
| 108 | Goa-1 | 3.69 | 3.03 | 3.42 | 3.38 | 31.27 | 31.22 | 33.4 | 31.96 |
| 109 | Gossypol free seed | 2.32 | 2.27 | 2.79 | 2.46 | 31.76 | 31.83 | 35.99 | 33.19 |
| 110 | GH-11-9-75 | 3.46 | 3.48 | 3.29 | 3.41 | 38.99 | 35.19 | 36.53 | 36.9 |
| 111 | Genetic Cotton | 4.11 | 3.35 | 3.15 | 3.54 | 37.8 | 37.72 | 38.78 | 38.1 |
| 112 | G.hir.S 2833 | 3.11 | 2.77 | 2.95 | 2.94 | 29.72 | 29.3 | 32.56 | 30.53 |
| 113 | Paymaster-111 | 3.45 | 2.7 | 3.33 | 3.16 | 33.31 | 33.57 | 37.22 | 34.7 |
| 114 | OK-86 | 3.22 | 2.66 | 3.12 | 3 | 32.94 | 32.9 | 34.86 | 33.57 |
| 115 | PAN F3 5575 | 2.95 | 3.32 | 3.03 | 3.1 | 36.97 | 35.25 | 34.59 | 35.6 |
| 116 | Northern . Star 4- 11 | 3.92 | 2.95 | 3.36 | 3.41 | 38.07 | 36.49 | 36.48 | 37.01 |
| 117 | Mysora American | 2.22 | 1.87 | 2.73 | 2.27 | 36.9 | 34.17 | 35.87 | 35.65 |
| 118 | Nectariless 73/33 | 2.39 | 2.51 | 3.01 | 2.64 | 34.38 | 35.35 | 37.36 | 35.7 |
| 119 | NCS-S-1 x P/1-1 | 3.13 | 3.12 | 3.2 | 3.15 | 34.4 | 31.45 | 34.7 | 33.52 |
| 120 | Mutant 189/75 | 3.41 | 2.97 | 3.28 | 3.22 | 38.54 | 34.19 | 33.52 | 35.42 |
| 121 | N-69 | 3.3 | 3.13 | 3.12 | 3.18 | 40.24 | 36.63 | 35.02 | 37.29 |
| 122 | HG-1 | 3.5 | 3.26 | 3.24 | 3.34 | 33.04 | 33.54 | 34.07 | 33.55 |
| 123 | MAR 444-2-70 | 3.71 | 3.21 | 3.2 | 3.38 | 35.05 | 34.62 | 35.02 | 34.9 |
| 124 | Huma-15 | 1.93 | 1.99 | 2.28 | 2.07 | 37.11 | 35.47 | 34.71 | 35.76 |
| 125 | HA-7 | 3.47 | 3.01 | 3.39 | 3.29 | 30.37 | 29.77 | 33.34 | 31.16 |
| 126 | H-SU-chow | 3.04 | 2.74 | 2.8 | 2.86 | 36.87 | 34.96 | 34.68 | 35.5 |
| 127 | H 2918-2 | 3.19 | 2.72 | 3.33 | 3.08 | 33.9 | 31.41 | 34.76 | 33.36 |
| 128 | Dos-056 | 2.15 | 2.28 | 2.33 | 2.25 | 38.42 | 36.05 | 35.59 | 36.69 |
| 129 | DPL-16 | 3.54 | 3.11 | 3.16 | 3.27 | 34.13 | 34.12 | 37.85 | 35.36 |
| 130 | Delcot2775 | 3.38 | 2.92 | 3.27 | 3.19 | 33.8 | 34.64 | 36.95 | 35.13 |
| 131 | Delfos 6-343-6 | 2.65 | 2.01 | 2.71 | 2.46 | 39.41 | 32.27 | 34.53 | 35.4 |
| 132 | DPL-SR-5 | 3.05 | 2.91 | 2.88 | 2.95 | 33.45 | 32.75 | 36.59 | 34.26 |
| 133 | DT –Webber | 3.98 | 3.71 | 3.32 | 3.67 | 37.96 | 34.25 | 34.3 | 35.5 |
| 134 | Coker 310(Fg) | 2.07 | 2.84 | 3.14 | 2.68 | 29.3 | 35.18 | 34.73 | 33.07 |
| 135 | Chung Mian | 2.74 | 2.41 | 3.02 | 2.72 | 35.42 | 35.82 | 35.7 | 35.65 |
| 136 | Co2-P1 | 3.56 | 3.21 | 3.27 | 3.35 | 38.95 | 36.83 | 36.42 | 37.4 |
| 137 | Cedix | 2.44 | 2.18 | 2.55 | 2.39 | 39.25 | 36.64 | 37 | 37.63 |
| 138 | Acala 1517 E | 3.3 | 2.92 | 3.25 | 3.16 | 39.21 | 38.25 | 36.33 | 37.93 |
| 139 | A33-57 | 3.54 | 2.66 | 3.22 | 3.14 | 31.42 | 29.74 | 33.11 | 31.42 |
| 140 | PGMB-3300 | 3.37 | 3.65 | 3.22 | 3.42 | 38.51 | 36.93 | 37.43 | 37.62 |
| 141 | PGMB-1779 | 1.78 | 2.22 | 2.33 | 2.11 | 36.28 | 36.71 | 37.01 | 36.67 |
| 142 | PGMB-1408 | 2.28 | 2.26 | 2.43 | 2.33 | 37.63 | 35.9 | 36.79 | 36.77 |
| 143 | PGMB-3100 | 2.18 | 1.98 | 2.31 | 2.16 | 37.3 | 35.34 | 36.61 | 36.42 |
| 144 | IR-NIBGE-3701 | 2.95 | 2.51 | 2.82 | 2.76 | 43.37 | 43.85 | 43.68 | 43.63 |
| 145 | IR-NIBGE-3 | 3.24 | 3.03 | 3.29 | 3.18 | 39.46 | 37.87 | 38.84 | 38.72 |
| 146 | IR-NIBGE-901 | 3.33 | 3.33 | 3.26 | 3.31 | 39.68 | 37.94 | 38.16 | 38.59 |
| 147 | PGMB-1523 | 3.13 | 3.27 | 3.08 | 3.16 | 38.19 | 37.48 | 38.23 | 37.97 |
| 148 | PGMB-1526 | 2.72 | 2.58 | 2.71 | 2.67 | 38.92 | 37.36 | 37.98 | 38.09 |
| 149 | GN-31 | 2.09 | 1.94 | 2.25 | 2.09 | 33.40 | 31.54 | 33.30 | 32.75 |
| 150 | GN-2085 | 1.70 | 2.41 | 2.71 | 2.27 | 31.59 | 31.38 | 32.55 | 31.84 |
| 151 | AA-802 | 3.19 | 2.87 | 3.06 | 3.04 | 41.69 | 39.99 | 40.84 | 40.84 |
| 152 | CEMB-1 | 2.09 | 2.04 | 2.33 | 2.15 | 37.79 | 36.98 | 37.76 | 37.51 |
| 153 | IR-NIBGE-1524 | 2.42 | 2.24 | 2.4 | 2.35 | 38.21 | 36.49 | 37.57 | 37.42 |
| 154 | AA-703 | 2.27 | 2.09 | 2.44 | 2.26 | 38.00 | 36.42 | 38.09 | 37.5 |
| 155 | FH-113 | 2.24 | 2.09 | 2.33 | 2.22 | 37.94 | 36.38 | 37.71 | 37.34 |
| 156 | CEMB-2 | 2.15 | 1.98 | 2.23 | 2.12 | 36.95 | 35.87 | 37.45 | 36.76 |
| 157 | Sitara-008 | 3.45 | 3.26 | 3.19 | 3.30 | 40.00 | 38.55 | 39.04 | 39.2 |
| 158 | MG-6 | 2.29 | 2.15 | 2.32 | 2.25 | 38.04 | 36.61 | 37.76 | 37.47 |
| 159 | Neelum-121 | 2.93 | 2.8 | 2.99 | 2.91 | 41.35 | 39.29 | 40.38 | 40.34 |
| 160 | Rohi | 2.22 | 2.06 | 2.34 | 2.2 | 18.72 | 17.22 | 17.84 | 17.92 |
| 161 | LDL-113 | 0.93 | 1.58 | 1.84 | 1.45 | 19.34 | 19.13 | 19.36 | 19.27 |
| 162 | No.18 | 1.66 | 1.42 | 1.58 | 1.55 | 28.37 | 26.72 | 27.54 | 27.54 |
| 163 | FDH-113 | 2.13 | 2.08 | 2.26 | 2.16 | 25.13 | 24.18 | 24.53 | 24.61 |
| 164 | No-36 | 2.11 | 1.97 | 2.15 | 2.07 | 24.44 | 23.13 | 23.56 | 23.71 |
| 165 | No-29 | 1.84 | 1.78 | 1.99 | 1.87 | 29.6 | 28.23 | 28.74 | 28.86 |
| 166 | 212 | 1.88 | 1.83 | 2.01 | 1.91 | 18.67 | 17.44 | 18.3 | 18.14 |
| 167 | Barnecum | 1.23 | 1.82 | 2.09 | 1.71 | 26.93 | 23.16 | 26.99 | 25.69 |
| 168 | FDH-786 | 2.27 | 2.26 | 2.29 | 2.27 | 28.16 | 26.99 | 27.49 | 27.54 |
| 169 | 451-R | 2.48 | 2.42 | 2.51 | 2.47 | 28.47 | 27.19 | 27.62 | 27.76 |
| 170 | 23718 | 2.42 | 2.4 | 2.49 | 2.44 | 18.49 | 17.2 | 18.05 | 17.91 |
| 171 | M-11 | 1.95 | 1.89 | 2.1 | 1.98 | 22.3 | 21.13 | 21.8 | 21.75 |
| 172 | Garohill | 1.18 | 1 | 1.24 | 1.14 | 21.85 | 20.99 | 21.42 | 21.42 |
| 173 | D-9 | 1.43 | 1.31 | 1.51 | 1.42 | 29.22 | 28.11 | 28.76 | 28.7 |
| 174 | HK-244 | 2.28 | 2.17 | 2.27 | 2.24 | 27.1 | 26.27 | 26.66 | 26.68 |
| 175 | M-17 | 2.41 | 2.33 | 2.46 | 2.4 | 23.51 | 21.99 | 22.86 | 22.79 |
| 176 | Synthetic | 2.26 | 2.22 | 2.22 | 2.23 | 24.68 | 23.47 | 24.04 | 24.06 |
| 177 | FDH-228 | 1.47 | 2.19 | 2.32 | 1.99 | 26.01 | 25.82 | 26.06 | 25.97 |
| 178 | No-17 | 2.3 | 2.27 | 2.40 | 2.32 | 36.84 | 35.54 | 36.00 | 36.13 |
| 179 | Haroonabad | 2.02 | 2.04 | 2.20 | 2.09 | 18.23 | 17.93 | 18.13 | 18.10 |
| 180 | 27518 | 1.98 | 2.04 | 2.19 | 2.07 | 18.14 | 17.68 | 18.5 | 18.10 |
| 181 | 786 | 1.93 | 1.84 | 2.07 | 1.95 | 27.85 | 26.61 | 26.88 | 27.11 |
| 182 | 231-R | 1.58 | 2.36 | 2.46 | 2.13 | 25.03 | 25.02 | 25.34 | 25.13 |
| 183 | 450-R | 1.97 | 1.89 | 2.08 | 1.98 | 20.39 | 20.23 | 20.52 | 20.38 |
| 184 | DC-116 | 1.67 | 2.35 | 2.53 | 2.18 | 25.22 | 25.11 | 25.14 | 25.16 |
| 185 | No-35 | 2.16 | 2.09 | 2.28 | 2.18 | 19.54 | 19.22 | 19.17 | 19.31 |
| Mean | | 2.8 | 2.55 | 2.83 | 2.73 | 34.31 | 33.03 | 33.8 | 33.71 |
| Minimum | | 0.93 | 1.00 | 1.24 | 1.14 | 18.14 | 17.2 | 17.84 | 17.91 |
| Maximum | | 4.11 | 3.71 | 3.77 | 3.67 | 43.37 | 3.85 | 43.68 | 43.63 |
| S.D | | 0.62 | 0.64 | 0.59 | 0.62 | 13.16 | 12.25 | 12.99 | 12.8 |
| S.E | | 0.05 | 0.05 | 0.04 | 0.05 | 0.97 | 0.90 | 0.96 | 0.94 |
| C.V | | 0.223 | 0.25 | 0.209 | 0.226 | 0.383 | 0.371 | 0.384 | 0.38 |
| C.D 0.05 | | 0.966 | 0.814 | 0.650 | 0.962 | 0.966 | 2.817 | 0.650 | 5.120 |

FSD=Faisalabad, VH=Vehari, MLN=Multan, S.D= Standard deviation, S.E=Standard error, C.V=Coefficient of variance, C.D=Critical difference

Supplementary Table 3: Mean performance of 185 cotton genotypes for micronaire value and staple length

| Sr # | Genotypes | Micronaire value (µg/inch) | | | | Staple length (mm) | | | |
| --- | --- | --- | --- | --- | --- | --- | --- | --- | --- |
|  |  | FSD | VH | MLN | Ave. | FSD | VH | MLN | Ave. |
| 1 | G.S/LB-602 | 4.82 | 5.57 | 5.04 | 5.14 | 27.91 | 28.85 | 28.41 | 28.39 |
| 2 | Lankart-57 | 4.77 | 5.61 | 4.34 | 4.91 | 27.93 | 29.05 | 28.55 | 28.51 |
| 3 | LB-546 | 4.76 | 5.17 | 4.69 | 4.87 | 26.64 | 26.89 | 26.95 | 26.83 |
| 4 | LAT 27-588-1740 | 5.50 | 5.62 | 5.44 | 5.52 | 25.84 | 26.71 | 28.02 | 26.86 |
| 5 | LB-391 | 4.13 | 5.23 | 3.84 | 4.4 | 26.18 | 26.09 | 26.25 | 26.17 |
| 6 | LA Okra 541 | 4.97 | 4.91 | 4.69 | 4.86 | 25.42 | 26.78 | 29.35 | 27.18 |
| 7 | Lankart | 4.05 | 5.02 | 4.19 | 4.42 | 27.87 | 29.53 | 26.92 | 28.11 |
| 8 | Lambright GLN(g1) | 5.24 | 5.49 | 4.84 | 5.19 | 24.5 | 25.46 | 26.35 | 25.43 |
| 9 | Lakburn | 4.00 | 4.76 | 3.74 | 4.17 | 24.34 | 25.02 | 25.85 | 25.07 |
| 10 | LA fregobract-2 | 5.35 | 6.03 | 5.34 | 5.57 | 24.89 | 25.65 | 26.62 | 25.72 |
| 11 | G.hir.138F | 3.91 | 4.93 | 3.84 | 4.23 | 23.69 | 25.85 | 21.75 | 23.76 |
| 12 | G 838 | 3.94 | 5 | 4.29 | 4.41 | 25.99 | 26.32 | 29.45 | 27.26 |
| 13 | FC 4245 | 4.76 | 4.94 | 5.04 | 4.91 | 24.46 | 25.61 | 27.52 | 25.86 |
| 14 | GAR F1 –M1 | 3.74 | 4.24 | 3.58 | 3.85 | 23.79 | 26.23 | 25.85 | 25.29 |
| 15 | F281 (g1) | 4.27 | 5.08 | 4.19 | 4.51 | 27.93 | 28.65 | 28.65 | 28.41 |
| 16 | Empire Hc-Pl | 5.17 | 5.49 | 4.64 | 5.1 | 27.69 | 28.03 | 28.82 | 28.18 |
| 17 | Early cot 31 | 5.15 | 4.64 | 4.69 | 4.83 | 23.93 | 26.27 | 25.72 | 25.31 |
| 18 | Dung Ding | 4.45 | 5.25 | 4.09 | 4.6 | 25.97 | 26.98 | 28.75 | 27.23 |
| 19 | E-302 | 4.46 | 4.97 | 4.34 | 4.59 | 25.76 | 26.99 | 27.82 | 26.86 |
| 20 | Dunn 120 | 5.34 | 5.86 | 5.24 | 5.48 | 26.92 | 28 | 27.25 | 27.39 |
| 21 | Paymaster-909 | 4.76 | 4.67 | 4.29 | 4.57 | 24.21 | 25.29 | 26.82 | 25.44 |
| 22 | PD 6520 | 5.28 | 5.15 | 4.49 | 4.97 | 23.08 | 24.76 | 26.95 | 24.93 |
| 23 | Peking Cotton | 4.45 | 5.72 | 4.03 | 4.74 | 24.17 | 26.19 | 27.22 | 25.86 |
| 24 | Pengze | 4.7 | 5.1 | 4.64 | 4.81 | 25.47 | 27.01 | 26.95 | 26.48 |
| 25 | Philippine Cotton | 4.42 | 4.28 | 4.66 | 4.46 | 25.41 | 26.84 | 29.22 | 27.16 |
| 26 | PRS – 72 | 3.6 | 4.46 | 3.67 | 3.91 | 25.5 | 24.91 | 27.75 | 26.05 |
| 27 | RA 31-9 | 4.93 | 4.63 | 4.54 | 4.7 | 24.87 | 26.25 | 27.05 | 26.06 |
| 28 | Bayou SM- 1 | 4.71 | 4.47 | 4.09 | 4.42 | 24.89 | 26.15 | 29.32 | 26.79 |
| 29 | Bar 96/40 | 4.07 | 4.38 | 3.84 | 4.1 | 23.33 | 24.57 | 25.85 | 24.58 |
| 30 | Belonga-5531 | 4.97 | 5.2 | 4.94 | 5.04 | 25.27 | 25.57 | 27.22 | 26.02 |
| 31 | Blanco 3363 | 3.00 | 3.52 | 3.49 | 3.34 | 26.82 | 28.1 | 28.75 | 27.89 |
| 32 | BjA-HL-27-B/163 | 5.48 | 5.78 | 5.44 | 5.57 | 26.18 | 27.07 | 31.45 | 28.23 |
| 33 | Blight master A-5 | 4.76 | 4.87 | 4.74 | 4.79 | 24.2 | 25.7 | 26.02 | 25.31 |
| 34 | Bambasa-49 | 4.34 | 5.31 | 4.49 | 4.71 | 25.27 | 26.51 | 29.15 | 26.98 |
| 35 | Meads | 4.62 | 6.19 | 4.74 | 5.18 | 24.64 | 25.55 | 27.72 | 25.97 |
| 36 | MCU-5 | 4.61 | 5.05 | 4.99 | 4.88 | 23.33 | 24.08 | 26.25 | 24.56 |
| 37 | MC-nair-220 | 5.47 | 5.31 | 5.14 | 5.31 | 25.35 | 27.22 | 29.35 | 27.31 |
| 38 | Mex-I | 4.81 | 5.05 | 4.24 | 4.7 | 26.57 | 28.19 | 30.42 | 28.39 |
| 39 | MC-nair-3150 | 4.81 | 5.08 | 4.09 | 4.66 | 26.76 | 27.27 | 28.25 | 27.43 |
| 40 | Malawi A637xK4219 | 4.79 | 4.9 | 3.95 | 4.55 | 27.12 | 26.84 | 27.92 | 27.29 |
| 41 | Locket-77 | 4.09 | 4.22 | 3.69 | 4 | 24.57 | 25.35 | 26.05 | 25.33 |
| 42 | M- 11 (G.hir) | 4.94 | 5.65 | 4.84 | 5.14 | 26.86 | 27.59 | 27.25 | 27.23 |
| 43 | Lumain No.1 | 4.77 | 5.6 | 5.24 | 5.2 | 26.18 | 28.44 | 31.52 | 28.71 |
| 44 | Delcot 277 NAF | 5.4 | 5.46 | 5.34 | 5.4 | 25.25 | 25.31 | 26.15 | 25.57 |
| 45 | Coker wild | 4.26 | 4.47 | 4.24 | 4.32 | 25.66 | 26.72 | 27.82 | 26.73 |
| 46 | Copal-10 | 5.08 | 6.07 | 5.04 | 5.4 | 25.13 | 24.95 | 28.85 | 26.31 |
| 47 | Culture 108-3 | 4.32 | 4.96 | 4.04 | 4.44 | 27.61 | 28.13 | 27.85 | 27.86 |
| 48 | Dai 16 | 5.24 | 5.9 | 4.99 | 5.38 | 23.63 | 24.69 | 26.02 | 24.78 |
| 49 | D-9 (G.hir) | 5.71 | 5.48 | 5.99 | 5.73 | 22.18 | 25.45 | 28.72 | 25.45 |
| 50 | D-3-75 | 5.05 | 4.32 | 4.09 | 4.49 | 23.62 | 25.57 | 27.35 | 25.51 |
| 51 | CA (68)36 | 5.82 | 5.49 | 4.94 | 5.42 | 23.67 | 25.88 | 30.45 | 26.67 |
| 52 | BW-76-31DH | 5.17 | 5.89 | 4.94 | 5.33 | 24.57 | 25.85 | 26.45 | 25.62 |
| 53 | B.T.K.12 | 4.6 | 4.78 | 5.09 | 4.82 | 24.93 | 27.15 | 29.52 | 27.2 |
| 54 | Brace 67B | 5.47 | 5.83 | 4.24 | 5.18 | 25.84 | 27.09 | 28.85 | 27.26 |
| 55 | BP-52 | 4.46 | 4.94 | 4.79 | 4.73 | 24.1 | 25.41 | 28.25 | 25.92 |
| 56 | Brace 81/6 | 4.8 | 5.44 | 4.44 | 4.89 | 24.93 | 26.69 | 27.32 | 26.32 |
| 57 | Bry cot-4 | 4.68 | 4.9 | 3.89 | 4.49 | 24.49 | 24.93 | 27.35 | 25.59 |
| 58 | BPA-66 | 5.6 | 5.61 | 5.54 | 5.58 | 26.48 | 27.44 | 28.52 | 27.48 |
| 59 | Carolina Queen (1) | 4.74 | 5.38 | 4.69 | 4.94 | 26.39 | 26.17 | 27.55 | 26.71 |
| 60 | Cascot B-2 | 5.32 | 5.05 | 5.19 | 5.19 | 23.04 | 24.08 | 26.45 | 24.52 |
| 61 | Al reba/202 | 5.14 | 5.57 | 5.24 | 5.32 | 24.9 | 26.9 | 28.45 | 26.75 |
| 62 | Albacala(69)32 | 4.83 | 4.9 | 5.14 | 4.96 | 25.46 | 25.54 | 26.65 | 25.88 |
| 63 | ALK 4371/468 | 4.94 | 5.21 | 4.34 | 4.83 | 25.47 | 26.81 | 28.65 | 26.97 |
| 64 | Albar A(57) 12 | 4.7 | 4.6 | 4.59 | 4.63 | 25.31 | 26.96 | 28.52 | 26.93 |
| 65 | Allepo-40 | 5.58 | 6.45 | 5.54 | 5.86 | 24.15 | 24.88 | 28.25 | 25.76 |
| 66 | AL-SR 1054-2/302 | 4.82 | 4.84 | 4.64 | 4.77 | 25.66 | 26.52 | 29.85 | 27.34 |
| 67 | Aldel-18 | 4.86 | 5.23 | 4.59 | 4.89 | 25.05 | 26.27 | 27.72 | 26.35 |
| 68 | B-799 | 4.34 | 5.67 | 4.94 | 4.98 | 26.42 | 28.17 | 31.25 | 28.61 |
| 69 | Arkugo c4 | 4.16 | 4.45 | 4.29 | 4.3 | 25.33 | 27.82 | 26.42 | 26.52 |
| 70 | Babdal | 5.02 | 4.99 | 4.44 | 4.82 | 24.45 | 25.4 | 27.15 | 25.66 |
| 71 | Arizona-6218 | 4.65 | 4.9 | 4.49 | 4.68 | 24.49 | 26.7 | 29.52 | 26.9 |
| 72 | A.T.H.783 | 4.8 | 5.46 | 4.54 | 4.93 | 27.7 | 28.26 | 29.45 | 28.47 |
| 73 | Austin | 5.65 | 4.43 | 5.79 | 5.29 | 24.79 | 25.86 | 27.32 | 25.99 |
| 74 | ASA(65)38 | 5.51 | 6.01 | 4.94 | 5.49 | 25.39 | 26.53 | 33.25 | 28.39 |
| 75 | AMSI-74 | 4.8 | 5.15 | 5.34 | 5.1 | 24.39 | 26.91 | 28.55 | 26.61 |
| 76 | ICA- 17NAF | 4.6 | 5.33 | 4.54 | 4.82 | 26.02 | 26.99 | 27.55 | 26.85 |
| 77 | IM-216 | 4.48 | 5.03 | 5.04 | 4.85 | 25.85 | 26.51 | 27.25 | 26.53 |
| 78 | Inta-SP-Toba-II | 4.78 | 4.42 | 4.39 | 4.53 | 24.57 | 27.34 | 26.92 | 26.28 |
| 79 | Imperial Acala | 4.43 | 4.8 | 4.44 | 4.56 | 25.81 | 28.06 | 29.85 | 27.91 |
| 80 | Kapal (A2106) | 4.69 | 5.09 | 4.44 | 4.74 | 24.7 | 26.57 | 28.25 | 26.51 |
| 81 | Karnak 55 | 4.33 | 4.9 | 4.19 | 4.47 | 27.85 | 27.56 | 29.22 | 28.21 |
| 82 | KapIana | 4.56 | 5.64 | 3.94 | 4.71 | 24.93 | 26.61 | 27.75 | 26.43 |
| 83 | Kuanung | 4.71 | 4.9 | 4.39 | 4.67 | 25.35 | 26.73 | 27.22 | 26.43 |
| 84 | L1-42-9 | 4.9 | 5.71 | 4.64 | 5.08 | 26.41 | 27.87 | 29.6 | 27.96 |
| 85 | Mex-13 | 4.69 | 5.44 | 3.69 | 4.61 | 25.94 | 27.5 | 27.72 | 27.05 |
| 86 | MG-H-NE-523 | 4.69 | 5.75 | 4.34 | 4.93 | 26.23 | 28.57 | 28.55 | 27.78 |
| 87 | MK-73 | 4.89 | 5.6 | 4.54 | 5.01 | 27.74 | 28.07 | 29.35 | 28.39 |
| 88 | Misdale | 5.14 | 5.18 | 5.54 | 5.29 | 26.02 | 28.13 | 28.62 | 27.59 |
| 89 | Muka (A641) | 4 | 5.18 | 3.79 | 4.32 | 26.45 | 28.03 | 24.25 | 26.24 |
| 90 | Mex-8 | 4.78 | 5.31 | 4.44 | 4.84 | 25.85 | 27.51 | 26.35 | 26.57 |
| 91 | Acala 1517-77 | 5.12 | 5.36 | 4.54 | 5.01 | 25.66 | 26.77 | 25.05 | 25.83 |
| 92 | AL-4-42/22 | 4.7 | 5.05 | 4.59 | 4.78 | 26.44 | 27.47 | 26.92 | 26.94 |
| 93 | Afghanistan-1 | 4.76 | 5.45 | 4.04 | 4.75 | 25.54 | 26.43 | 31.45 | 27.8 |
| 94 | AET-5 | 4.46 | 4.93 | 4.39 | 4.59 | 22.62 | 24.46 | 26.35 | 24.48 |
| 95 | Accopaymaster | 5.03 | 5.1 | 5.19 | 5.11 | 22.98 | 23.86 | 24.45 | 23.76 |
| 96 | AH( 67)M | 4.96 | 5.49 | 5.14 | 5.2 | 26.07 | 26.47 | 27.85 | 26.8 |
| 97 | IAC- 12 | 5.09 | 5.73 | 5.14 | 5.32 | 26.68 | 28.05 | 30.85 | 28.53 |
| 98 | Hybe 201 | 4.87 | 4.35 | 4.29 | 4.5 | 26.7 | 26.25 | 26.92 | 26.62 |
| 99 | HL-1 | 5.14 | 5.11 | 4.74 | 5.00 | 26.19 | 27.4 | 28.45 | 27.34 |
| 100 | Hopiacala | 4.95 | 5.11 | 4.79 | 4.95 | 24.07 | 27.14 | 26.42 | 25.88 |
| 101 | High gossypol germplasm | 5.51 | 5.6 | 5.34 | 5.48 | 23.77 | 27.16 | 29.35 | 26.76 |
| 102 | H-496 | 4.49 | 5.14 | 3.99 | 4.54 | 27.26 | 26.98 | 26.82 | 27.02 |
| 103 | HG-18-45-N | 4.27 | 4.37 | 4.44 | 4.36 | 25.82 | 26.37 | 26.25 | 26.14 |
| 104 | G.Var.Coker | 4.72 | 4.58 | 3.39 | 4.23 | 23.95 | 26.37 | 27.82 | 26.05 |
| 105 | Gumbo Okra | 4.89 | 4.86 | 5.34 | 5.03 | 26.22 | 27.1 | 28.65 | 27.32 |
| 106 | GSA-71 | 4.28 | 5.61 | 4.24 | 4.71 | 25.4 | 27.15 | 28.25 | 26.93 |
| 107 | Greeg 25V | 5.07 | 4.94 | 4.94 | 4.98 | 25.56 | 26.91 | 27.75 | 26.74 |
| 108 | Goa-1 | 4.68 | 4.79 | 4.24 | 4.57 | 25.9 | 26.3 | 26.65 | 26.28 |
| 109 | Gossypol free seed | 4.39 | 4.36 | 3.79 | 4.18 | 26.76 | 27.44 | 29.32 | 27.84 |
| 110 | GH-11-9-75 | 4.64 | 5.1 | 4.84 | 4.86 | 26.36 | 26.88 | 27.55 | 26.93 |
| 111 | Genetic Cotton | 4.68 | 5.22 | 4.99 | 4.96 | 26.43 | 27.37 | 26.32 | 26.71 |
| 112 | G.hir.S 2833 | 4.69 | 4.81 | 4.84 | 4.78 | 27.71 | 27.56 | 26.85 | 27.37 |
| 113 | Paymaster-111 | 4.71 | 5.21 | 5.04 | 4.99 | 26.41 | 28.18 | 26.55 | 27.05 |
| 114 | OK-86 | 5.39 | 5.4 | 4.74 | 5.18 | 25.52 | 26.45 | 26.75 | 26.24 |
| 115 | PAN F3 5575 | 4.42 | 4.36 | 3.89 | 4.22 | 27.88 | 29.95 | 32.82 | 30.22 |
| 116 | Northern. Star 4- 11 | 5.44 | 5.64 | 5.34 | 5.47 | 24.46 | 26.27 | 26.92 | 25.88 |
| 117 | Mysora American | 5.20 | 4.96 | 4.09 | 4.75 | 22.94 | 23.95 | 23.55 | 23.48 |
| 118 | Nectariless 73/33 | 5.6 | 5.28 | 5.59 | 5.49 | 22.52 | 23.27 | 26.45 | 24.08 |
| 119 | NCS-S-1 x P/1-1 | 5.34 | 5.17 | 4.54 | 5.02 | 24.51 | 25.24 | 24.85 | 24.87 |
| 120 | Mutant 189/75 | 4.9 | 4.74 | 4.44 | 4.69 | 26.66 | 28.3 | 30.55 | 28.5 |
| 121 | N-69 | 5.44 | 6.14 | 4.34 | 5.31 | 26.33 | 26.48 | 29.15 | 27.32 |
| 122 | HG-1 | 5.08 | 5.31 | 4.84 | 5.08 | 27.94 | 29.45 | 29.05 | 28.81 |
| 123 | MAR 444-2-70 | 5.05 | 4.91 | 4.34 | 4.77 | 26.53 | 27.01 | 28.15 | 27.23 |
| 124 | Huma-15 | 5.19 | 4.96 | 3.39 | 4.51 | 26.05 | 26.84 | 28.62 | 27.17 |
| 125 | HA-7 | 4.66 | 6.27 | 4.34 | 5.09 | 27.65 | 28.71 | 27.95 | 28.1 |
| 126 | H-SU-chow | 4.77 | 6.2 | 3.94 | 4.97 | 24.69 | 26.66 | 26.8 | 26.05 |
| 127 | H 2918-2 | 4.71 | 4.57 | 3.94 | 4.41 | 25.57 | 25.88 | 24.85 | 25.43 |
| 128 | Dos-056 | 5.21 | 5.06 | 4.79 | 5.02 | 25.56 | 26.66 | 29.02 | 27.08 |
| 129 | DPL-16 | 4.76 | 5.14 | 5.14 | 5.01 | 24.92 | 27.85 | 28.65 | 27.14 |
| 130 | Delcot2775 | 4.97 | 5.67 | 6.04 | 5.56 | 27.9 | 27.47 | 30.45 | 28.6 |
| 131 | Delfos 6-343-6 | 5.23 | 5.12 | 4.24 | 4.86 | 26.05 | 27.94 | 29.12 | 27.71 |
| 132 | DPL-SR-5 | 5.01 | 5.21 | 5.44 | 5.22 | 25.8 | 26.9 | 28.25 | 26.98 |
| 133 | DT-Webber | 5.53 | 5.45 | 4.34 | 5.11 | 26.67 | 27.95 | 29.85 | 28.15 |
| 134 | Coker 310(Fg) | 4.89 | 5.36 | 3.99 | 4.75 | 25.01 | 27.08 | 29.72 | 27.27 |
| 135 | Chung Mian | 5.19 | 5.95 | 5.14 | 5.43 | 24.86 | 25.48 | 27.35 | 25.89 |
| 136 | Co2-P1 | 4.85 | 4.96 | 4.34 | 4.72 | 24.97 | 26.56 | 28.25 | 26.59 |
| 137 | Cedix | 5.12 | 5.20 | 5.39 | 5.24 | 24.24 | 26.31 | 28.42 | 26.32 |
| 138 | Acala 1517 E | 4.82 | 5.35 | 4.64 | 4.94 | 25.3 | 27.47 | 26.75 | 26.50 |
| 139 | A33-57 | 4.82 | 6.20 | 4.34 | 5.12 | 26.07 | 27.26 | 28.05 | 27.13 |
| 140 | PGMB-3300 | 4.95 | 5.31 | 5.31 | 5.19 | 30.17 | 29.88 | 30.46 | 30.17 |
| 141 | PGMB-1779 | 4.15 | 5.31 | 4.55 | 4.67 | 27.81 | 27.83 | 29.08 | 28.24 |
| 142 | PGMB-1408 | 4.92 | 4.66 | 5.16 | 4.91 | 28.44 | 29.00 | 29.41 | 28.95 |
| 143 | PGMB-3100 | 5.04 | 5.34 | 5.57 | 5.32 | 26.92 | 27.31 | 28.14 | 27.46 |
| 144 | IR-NIBGE-3701 | 4.66 | 5.08 | 5.10 | 4.95 | 28.45 | 27.14 | 28.28 | 27.96 |
| 145 | IR-NIBGE-3 | 5.34 | 5.22 | 5.18 | 5.25 | 28.88 | 28.93 | 29.81 | 29.20 |
| 146 | IR-NIBGE-901 | 5.62 | 5.64 | 5.5 | 5.59 | 26.82 | 27.91 | 28.95 | 27.89 |
| 147 | PGMB-1523 | 4.86 | 4.66 | 4.61 | 4.71 | 27.85 | 28.22 | 28.81 | 28.29 |
| 148 | PGMB-1526 | 4.9 | 4.96 | 4.94 | 4.93 | 24.97 | 25.59 | 25.66 | 25.41 |
| 149 | GN-31 | 4.68 | 4.56 | 4.55 | 4.6 | 27.28 | 26.51 | 26.97 | 26.92 |
| 150 | GN-2085 | 5.02 | 5.08 | 5 | 5.03 | 28.82 | 29.2 | 29.62 | 29.21 |
| 151 | AA-802 | 4.67 | 4.39 | 4.32 | 4.46 | 28.14 | 27.84 | 29.52 | 28.5 |
| 152 | CEMB-1 | 5.28 | 5.15 | 5.06 | 5.16 | 26.66 | 27.15 | 28.09 | 27.3 |
| 153 | IR-NIBGE-1524 | 4.56 | 4.47 | 4.52 | 4.52 | 28.33 | 28.59 | 29.53 | 28.82 |
| 154 | AA-703 | 5.21 | 5.19 | 5.21 | 5.2 | 28.72 | 29.08 | 29.52 | 29.1 |
| 155 | FH-113 | 4.86 | 4.6 | 4.58 | 4.68 | 26.79 | 26.69 | 27.01 | 26.83 |
| 156 | CEMB-2 | 5.4 | 4.7 | 4.9 | 5 | 25.65 | 26.97 | 26.97 | 26.53 |
| 157 | Sitara-008 | 4.91 | 5.13 | 4.93 | 4.99 | 28.62 | 28.88 | 29.77 | 29.09 |
| 158 | MG-6 | 5.42 | 5.31 | 5.43 | 5.39 | 27.89 | 28.23 | 29.19 | 28.43 |
| 159 | Neelum-121 | 4.67 | 4.58 | 4.59 | 4.61 | 28.97 | 29.06 | 29.44 | 29.16 |
| 160 | Rohi | 4.64 | 5.19 | 4.71 | 4.85 | 16.97 | 17.2 | 17.52 | 17.23 |
| 161 | LDL-113 | 4.89 | 5.06 | 4.91 | 4.95 | 17.66 | 17.54 | 17.86 | 17.68 |
| 162 | No.18 | 4.99 | 5.01 | 4.96 | 4.99 | 15.22 | 15.17 | 15.63 | 15.34 |
| 163 | FDH-113 | 5.64 | 5.72 | 5.91 | 5.76 | 14.81 | 14.91 | 15.04 | 14.92 |
| 164 | No-36 | 5.44 | 5.58 | 5.5 | 5.51 | 13.86 | 13.52 | 13.92 | 13.76 |
| 165 | No-29 | 5.44 | 5.47 | 5.51 | 5.47 | 17.79 | 17.55 | 17.99 | 17.78 |
| 166 | 212 | 5.29 | 5 | 5.12 | 5.14 | 18.02 | 18.35 | 18.44 | 18.27 |
| 167 | Barnecum | 4.29 | 4.6 | 4.66 | 4.52 | 15.81 | 15.74 | 16.26 | 15.93 |
| 168 | FDH-786 | 5.64 | 5.73 | 5.61 | 5.66 | 15.42 | 15.57 | 16.04 | 15.67 |
| 169 | 451-R | 5.44 | 5.61 | 5.45 | 5.5 | 15.21 | 14.89 | 15.51 | 15.2 |
| 170 | 23718 | 4.86 | 4.88 | 4.95 | 4.9 | 13.98 | 14.86 | 14.17 | 14.33 |
| 171 | M-11 | 5.24 | 5.27 | 5.37 | 5.29 | 17.59 | 17.72 | 18.12 | 17.81 |
| 172 | Garohill | 5.09 | 5.22 | 5.12 | 5.14 | 18.12 | 18.19 | 18.22 | 18.18 |
| 173 | D-9 | 6.49 | 6.08 | 6.02 | 6.2 | 15.27 | 15.27 | 15.65 | 15.4 |
| 174 | HK-244 | 4.64 | 4.77 | 4.62 | 4.68 | 16.67 | 17 | 17.13 | 16.93 |
| 175 | M-17 | 5.44 | 5.55 | 5.45 | 5.48 | 14.57 | 14.99 | 15.18 | 14.91 |
| 176 | Synthetic | 4.09 | 4.32 | 4.2 | 4.2 | 23.94 | 23.83 | 24.29 | 24.02 |
| 177 | FDH-228 | 5.89 | 5.86 | 5.87 | 5.87 | 18.06 | 18.92 | 18.79 | 18.59 |
| 178 | No-17 | 6.04 | 6.03 | 6.05 | 6.04 | 19.23 | 19.45 | 19.75 | 19.48 |
| 179 | Haroonabad | 5.04 | 5.12 | 5.13 | 5.1 | 20.19 | 20.95 | 20.88 | 20.67 |
| 180 | 27518 | 5.16 | 5.16 | 5.29 | 5.2 | 17.56 | 17.7 | 17.99 | 17.75 |
| 181 | 786 | 4.84 | 4.99 | 4.91 | 4.91 | 13.46 | 14.05 | 14.19 | 13.9 |
| 182 | 231-R | 6.09 | 6.06 | 6.12 | 6.09 | 14.75 | 14.98 | 15.5 | 15.07 |
| 183 | 450-R | 5.27 | 5.25 | 5.37 | 5.3 | 20.16 | 20.37 | 20.53 | 20.35 |
| 184 | DC-116 | 5.49 | 5.47 | 5.57 | 5.51 | 13.08 | 13.18 | 13.9 | 13.39 |
| 185 | No-35 | 5.2 | 5.19 | 5.28 | 5.22 | 13.7 | 14.34 | 14.51 | 14.18 |
| Mean | | 4.89 | 5.17 | 4.73 | 4.93 | 24.5 | 25.46 | 26.45 | 25.47 |
| Minimum | | 3.00 | 3.52 | 3.39 | 3.34 | 13.08 | 13.18 | 13.9 | 13.39 |
| Maximum | | 6.49 | 6.45 | 6.12 | 6.20 | 30.17 | 29.95 | 33.25 | 30.22 |
| S.D | | 0.27 | 0.27 | 0.17 | 0.06 | 10.05 | 10.26 | 9.83 | 10.05 |
| S.E | | 0.02 | 0.02 | 0.01 | 0.00 | 0.74 | 0.75 | 0.72 | 0.74 |
| C.V | | 0.055 | 0.052 | 0.036 | 0.012 | 0.41 | 0.403 | 0.372 | 0.394 |
| C.D 0.05 | | 0.731 | 0.916 | 1.724 | 1.253 | 0.667 | 1.315 | 0.838 | 3.492 |

FSD=Faisalabad, VH=Vehari, MLN=Multan, S.D= Standard deviation, S.E=Standard error, C.V=Coefficient of variation, C.D=Critical difference

Supplementary Table 4 Mean performance of 185 cotton genotypes for fiber bundle strength and uniformity index

| Sr # | Genotypes | Fiber bundle strength (g/tex) | | | | Uniformity index (%) | | | |
| --- | --- | --- | --- | --- | --- | --- | --- | --- | --- |
|  |  | FSD | VH | MLN | Ave. | FSD | VH | MLN | Ave. |
| 1 | G.S/LB-602 | 29.19 | 31.34 | 31.1 | 30.54 | 81.23 | 80.03 | 81.12 | 80.79 |
| 2 | Lankart-57 | 27.04 | 28.7 | 30.18 | 28.64 | 81.78 | 82.29 | 83.92 | 82.66 |
| 3 | LB-546 | 29.42 | 29.34 | 29.69 | 29.48 | 81.36 | 82.81 | 84.06 | 82.74 |
| 4 | LAT 27-588-1740 | 27.95 | 31.93 | 31.23 | 30.37 | 81.78 | 81.18 | 82.63 | 81.86 |
| 5 | LB-391 | 28.63 | 26.63 | 28.38 | 27.88 | 79.51 | 80.43 | 81.76 | 80.56 |
| 6 | LA Okra 541 | 24.54 | 30.28 | 31.12 | 28.65 | 79.33 | 80.81 | 82.17 | 80.77 |
| 7 | Lankart | 27.35 | 30.77 | 31.24 | 29.78 | 80.23 | 80.73 | 82.97 | 81.31 |
| 8 | Lambright GLN(g1) | 26.24 | 28.49 | 30.09 | 28.27 | 79.18 | 79.88 | 80.67 | 79.91 |
| 9 | Lakburn | 24.83 | 28.11 | 28.48 | 27.14 | 77.81 | 79.03 | 80.23 | 79.02 |
| 10 | LA fregobract-2 | 22.8 | 27.69 | 25.83 | 25.44 | 78.43 | 79.55 | 80.88 | 79.62 |
| 11 | G.hir.138F | 22.78 | 29.52 | 27.66 | 26.65 | 77.16 | 79.58 | 82.27 | 79.67 |
| 12 | G 838 | 28.08 | 28.73 | 29.38 | 28.73 | 78.81 | 80.12 | 82.17 | 80.36 |
| 13 | FC 4245 | 24.9 | 27.32 | 28.79 | 27 | 77.23 | 78.52 | 79.74 | 78.5 |
| 14 | GAR F1 –M1 | 22.33 | 28.04 | 29.46 | 26.61 | 75.76 | 78.53 | 84.07 | 79.45 |
| 15 | F281 (g1) | 29.43 | 30.06 | 29.16 | 29.55 | 79.81 | 80.75 | 82.36 | 80.97 |
| 16 | Empire Hc-Pl | 24 | 29.49 | 28.37 | 27.29 | 79.98 | 80.23 | 81.23 | 80.48 |
| 17 | Early cot 31 | 24.75 | 28.01 | 29.73 | 27.5 | 79.08 | 79.57 | 80.46 | 79.7 |
| 18 | Dung Ding | 25.98 | 29.42 | 29.86 | 28.42 | 79.91 | 78.46 | 79.96 | 79.44 |
| 19 | E-302 | 24.25 | 26.31 | 28.73 | 26.43 | 78.88 | 78.61 | 79.6 | 79.03 |
| 20 | Dunn 120 | 27.54 | 29.33 | 30.1 | 28.99 | 80.2 | 80.48 | 82.68 | 81.12 |
| 21 | Paymaster-909 | 26.2 | 27.94 | 28.63 | 27.59 | 79.88 | 78.8 | 80.49 | 79.72 |
| 22 | PD 6520 | 22.73 | 27.73 | 28.82 | 26.43 | 77.26 | 77.88 | 78.56 | 77.9 |
| 23 | Peking Cotton | 25.7 | 27.42 | 29.04 | 27.38 | 79.23 | 80.75 | 82.17 | 80.72 |
| 24 | Pengze | 23.94 | 29.98 | 30.55 | 28.16 | 78.7 | 79.73 | 82.18 | 80.2 |
| 25 | Philippine Cotton | 24.3 | 28.3 | 29.65 | 27.42 | 78.33 | 79.94 | 80.51 | 79.59 |
| 26 | PRS-72 | 23.58 | 25.83 | 28.17 | 25.86 | 77.61 | 78.87 | 77.16 | 77.88 |
| 27 | RA 31-9 | 23.34 | 30.28 | 26.42 | 26.68 | 77.98 | 80.63 | 81.32 | 79.98 |
| 28 | Bayou SM- 1 | 22.65 | 29.33 | 28.53 | 26.84 | 77.53 | 78.75 | 80.14 | 78.81 |
| 29 | Bar 96/40 | 22.37 | 27.44 | 28.75 | 26.19 | 77.11 | 78.73 | 79.52 | 78.45 |
| 30 | Belonga-5531 | 21.45 | 25.9 | 27.64 | 25 | 77.93 | 78.26 | 78.88 | 78.36 |
| 31 | Blanco 3363 | 27.28 | 29.21 | 28.9 | 28.46 | 77.91 | 79.23 | 80.48 | 79.2 |
| 32 | BjA-HL-27-B/163 | 25.14 | 28.39 | 29.81 | 27.78 | 79.43 | 80.73 | 82.22 | 80.79 |
| 33 | Blight master A-5 | 21.45 | 26.73 | 27.71 | 25.3 | 76.68 | 78.8 | 79.46 | 78.31 |
| 34 | Bambasa-49 | 24.23 | 25.83 | 27.36 | 25.81 | 77.91 | 77.94 | 79.87 | 78.57 |
| 35 | Meads | 23.5 | 25.9 | 26.97 | 25.46 | 77.13 | 77.75 | 78.48 | 77.78 |
| 36 | MCU-5 | 24.08 | 29.03 | 27.77 | 26.96 | 79.61 | 80.94 | 82.57 | 81.04 |
| 37 | MC-nair-220 | 24.14 | 27.03 | 28.83 | 26.67 | 80.23 | 81.08 | 81.38 | 80.9 |
| 38 | Mex-I | 26.2 | 30.53 | 27.23 | 27.99 | 79.78 | 80.79 | 82.27 | 80.95 |
| 39 | MC-nair-3150 | 25.18 | 27.43 | 28.24 | 26.95 | 78.99 | 79.14 | 79.85 | 79.33 |
| 40 | Malawi A637xK4219 | 21.72 | 30.2 | 30.54 | 27.49 | 79.86 | 80.05 | 80.77 | 80.23 |
| 41 | Locket-77 | 24.33 | 28.25 | 29.37 | 27.32 | 77.51 | 78.36 | 79.43 | 78.43 |
| 42 | M- 11 (G.hir) | 24.24 | 30.49 | 29.92 | 28.22 | 77.88 | 79.08 | 79.9 | 78.95 |
| 43 | Lumain No.1 | 25.5 | 31.4 | 30.93 | 29.28 | 77.83 | 78.4 | 80.33 | 78.85 |
| 44 | Delcot 277 NAF | 26.24 | 29.6 | 30 | 28.61 | 79.38 | 80.46 | 81.97 | 80.6 |
| 45 | Coker wild | 24.19 | 26.81 | 28.7 | 26.57 | 77.79 | 78.9 | 79.47 | 78.72 |
| 46 | Copal-10 | 26.04 | 30.38 | 29.3 | 28.57 | 80.68 | 80.04 | 80.72 | 80.48 |
| 47 | Culture 108-3 | 25.64 | 28.03 | 29.6 | 27.76 | 79.53 | 80.13 | 80.38 | 80.01 |
| 48 | Dai 16 | 21.35 | 26.4 | 28.62 | 25.46 | 78.13 | 78.59 | 79.53 | 78.75 |
| 49 | D-9 (G.hir) | 24.69 | 29.31 | 30.2 | 28.06 | 77.48 | 78.58 | 79.78 | 78.61 |
| 50 | D-3-75 | 21.88 | 29.13 | 28.62 | 26.54 | 77.71 | 78.89 | 80.16 | 78.92 |
| 51 | CA (68)36 | 27.29 | 31.54 | 30.12 | 29.65 | 79.03 | 79.59 | 81.62 | 80.08 |
| 52 | BW-76-31DH | 24.54 | 30 | 29.69 | 28.08 | 79.73 | 80.47 | 81.87 | 80.69 |
| 53 | B.T.K.12 | 24.9 | 31.1 | 28.45 | 28.15 | 78.08 | 79.06 | 79.79 | 78.98 |
| 54 | Brace 67B | 25.29 | 30.82 | 29.68 | 28.6 | 79.28 | 80.48 | 80.96 | 80.24 |
| 55 | BP-52 | 22.38 | 30.84 | 28.33 | 27.18 | 77.66 | 80.08 | 79.82 | 79.19 |
| 56 | Brace 81/6 | 26.1 | 29.85 | 30.24 | 28.73 | 79.18 | 78.43 | 79.79 | 79.13 |
| 57 | Bry cot-4 | 25.08 | 30.64 | 29.38 | 28.36 | 79.66 | 80.24 | 80.37 | 80.09 |
| 58 | BPA-66 | 27.9 | 30.65 | 30.2 | 29.58 | 80.42 | 78.84 | 79.31 | 79.52 |
| 59 | Carolina Queen (1) | 26.28 | 27.63 | 28.38 | 27.43 | 80.34 | 79.1 | 79.43 | 79.62 |
| 60 | Cascot B-2 | 20.98 | 28.03 | 28.89 | 25.97 | 77.41 | 78.32 | 79.06 | 78.26 |
| 61 | Al reba/202 | 22.34 | 29.79 | 30.14 | 27.42 | 78.63 | 79.36 | 80.36 | 79.45 |
| 62 | Albacala(69)32 | 23.19 | 29.69 | 30.13 | 27.67 | 77.18 | 78.08 | 78.73 | 77.99 |
| 63 | ALK 4371/468 | 21.24 | 28.07 | 26.74 | 25.35 | 78.33 | 79.49 | 81.97 | 79.93 |
| 64 | Albar A(57) 12 | 24.9 | 30.14 | 28.93 | 27.99 | 77.88 | 78.46 | 79.2 | 78.51 |
| 65 | Allepo-40 | 25.49 | 27.89 | 29.14 | 27.51 | 79.93 | 79.49 | 79.87 | 79.76 |
| 66 | AL-SR 1054-2/302 | 22.89 | 30.68 | 28.3 | 27.29 | 78.88 | 80.16 | 80.9 | 79.98 |
| 67 | Aldel-18 | 25.2 | 30.77 | 29.2 | 28.39 | 80.23 | 80.3 | 80.85 | 80.46 |
| 68 | B-799 | 24.29 | 29.3 | 30.15 | 27.91 | 79.03 | 79.74 | 80.85 | 79.87 |
| 69 | Arkugo c4 | 28.2 | 30.38 | 29.24 | 29.27 | 78.93 | 80.13 | 80.34 | 79.8 |
| 70 | Babdal | 27.14 | 29.02 | 28.79 | 28.32 | 78.44 | 79.38 | 80.27 | 79.36 |
| 71 | Arizona-6218 | 22.2 | 29.1 | 29.76 | 27.02 | 77.98 | 78.31 | 78.78 | 78.36 |
| 72 | A.T.H.783 | 26.49 | 30.25 | 28.62 | 28.46 | 80.38 | 80.84 | 82.57 | 81.26 |
| 73 | Austin | 23.4 | 29.65 | 30.24 | 27.76 | 78.93 | 79.41 | 80.3 | 79.55 |
| 74 | ASA(65)38 | 22.59 | 28.9 | 29.65 | 27.05 | 79.83 | 80.37 | 80.74 | 80.31 |
| 75 | AMSI-74 | 23.44 | 28.23 | 29.71 | 27.13 | 78.13 | 79.33 | 80.25 | 79.23 |
| 76 | ICA- 17NAF | 24.49 | 30.7 | 28.92 | 28.04 | 79.28 | 80.35 | 80.38 | 80 |
| 77 | IM-216 | 28.09 | 29.51 | 30.13 | 29.24 | 80.08 | 80.95 | 80.53 | 80.52 |
| 78 | Inta-SP-Toba-II | 25.4 | 30.69 | 28.83 | 28.31 | 79.43 | 78.91 | 79.63 | 79.32 |
| 79 | Imperial Acala | 26.84 | 29.2 | 30.03 | 28.69 | 79.28 | 79.86 | 80.77 | 79.97 |
| 80 | Kapal (A2106) | 22.69 | 27.8 | 28.62 | 26.37 | 77.78 | 78.95 | 80.5 | 79.08 |
| 81 | Karnak 55 | 26.55 | 29.4 | 30.24 | 28.73 | 78.48 | 78.93 | 80.08 | 79.16 |
| 82 | KapIana | 24.64 | 29 | 29.68 | 27.77 | 77.88 | 78.88 | 80.62 | 79.12 |
| 83 | Kuanung | 26.25 | 29.15 | 30.26 | 28.55 | 78.43 | 79.85 | 82.27 | 80.18 |
| 84 | L1-42-9 | 28.49 | 30.68 | 30.02 | 29.73 | 80.93 | 80.79 | 81.81 | 81.17 |
| 85 | Mex-13 | 22.9 | 25.1 | 28.79 | 25.6 | 78.33 | 78.85 | 79.28 | 78.82 |
| 86 | MG-H-NE-523 | 24.44 | 30.3 | 28.62 | 27.79 | 79.73 | 80.56 | 81.77 | 80.69 |
| 87 | MK-73 | 27.39 | 30.3 | 29.08 | 28.92 | 80.48 | 81.23 | 81.38 | 81.03 |
| 88 | Misdale | 23.05 | 25.7 | 27.93 | 25.56 | 80.08 | 80.45 | 81.13 | 80.55 |
| 89 | Muka (A641) | 26.83 | 29.83 | 28.44 | 28.37 | 80.11 | 79.9 | 80.59 | 80.2 |
| 90 | Mex-8 | 27.89 | 28.10 | 29.92 | 28.64 | 79.88 | 79.18 | 80.89 | 79.98 |
| 91 | Acala 1517-77 | 25.39 | 29.90 | 28.92 | 28.07 | 79.53 | 80.47 | 81.32 | 80.44 |
| 92 | AL-4-42/22 | 26.15 | 30.90 | 28.73 | 28.59 | 80.5 | 79.43 | 80.3 | 80.08 |
| 93 | Afghanistan-1 | 23.19 | 30.00 | 29.13 | 27.44 | 78.73 | 79.4 | 82.02 | 80.05 |
| 94 | AET – 5 | 25.63 | 29.73 | 29.03 | 28.13 | 77.31 | 77.99 | 80.76 | 78.69 |
| 95 | Accopaymaster | 23.23 | 26.93 | 27.92 | 26.03 | 77.21 | 79.09 | 80.56 | 78.95 |
| 96 | AH( 67)M | 28.06 | 29.20 | 30.09 | 29.12 | 81.21 | 80.44 | 81.39 | 81.01 |
| 97 | IAC- 12 | 27.64 | 29.96 | 29.93 | 29.18 | 81.03 | 80.27 | 81.76 | 81.02 |
| 98 | Hybe 201 | 24.00 | 30.2 | 30.61 | 28.27 | 80.38 | 80.53 | 80.77 | 80.56 |
| 99 | HL-1 | 30.04 | 30.05 | 29.49 | 29.86 | 79.88 | 80.45 | 80.93 | 80.42 |
| 100 | Hopiacala | 22.45 | 30.9 | 28.85 | 27.4 | 78.13 | 78.43 | 79.44 | 78.67 |
| 101 | High gossypol germplasm | 23.19 | 30.5 | 26.24 | 26.64 | 77.33 | 78.76 | 80.39 | 78.82 |
| 102 | H-496 | 25.95 | 27.6 | 28.8 | 27.45 | 78.68 | 80.18 | 81.57 | 80.14 |
| 103 | HG-18-45-N | 23.24 | 26.62 | 28.01 | 25.96 | 76.88 | 78.17 | 79.52 | 78.19 |
| 104 | G.Var.Coker | 24.25 | 27.51 | 28.23 | 26.66 | 77.78 | 79.4 | 78.44 | 78.54 |
| 105 | Gumbo Okra | 22.16 | 30.7 | 29.08 | 27.31 | 78.65 | 79.39 | 81.92 | 79.99 |
| 106 | GSA-71 | 23.94 | 29.7 | 28.42 | 27.35 | 77.23 | 79.39 | 80.38 | 79.00 |
| 107 | Greeg 25V | 21.99 | 26.21 | 28.03 | 25.41 | 78.73 | 80.00 | 80.32 | 79.68 |
| 108 | Goa-1 | 23.14 | 27.06 | 28.98 | 26.39 | 78.68 | 79.47 | 80.49 | 79.55 |
| 109 | Gossypol free seed | 24.9 | 30.56 | 28.63 | 28.03 | 77.58 | 78.91 | 79.4 | 78.63 |
| 110 | GH-11-9-75 | 26.09 | 29.8 | 30.12 | 28.67 | 78.68 | 79.75 | 80.62 | 79.68 |
| 111 | Genetic Cotton | 23.95 | 30.5 | 28.64 | 27.7 | 76.58 | 78.45 | 80.53 | 78.52 |
| 112 | G.hir.S 2833 | 27.79 | 25.64 | 29.95 | 27.79 | 80.93 | 79.86 | 80.36 | 80.38 |
| 113 | Paymaster-111 | 25.39 | 30.1 | 30.52 | 28.67 | 78.78 | 79.65 | 80.92 | 79.78 |
| 114 | OK-86 | 23.44 | 29.9 | 30.51 | 27.95 | 79.23 | 80.05 | 80.85 | 80.04 |
| 115 | PAN F3 5575 | 28.3 | 29.12 | 30.33 | 29.25 | 79.83 | 79.28 | 79.48 | 79.53 |
| 116 | Northern . Star 4- 11 | 24.9 | 29.45 | 30.23 | 28.19 | 79.23 | 80.48 | 81.33 | 80.35 |
| 117 | Mysora American | 23.73 | 28.31 | 29.27 | 27.1 | 77.06 | 76.92 | 78.31 | 77.43 |
| 118 | Nectariless 73/33 | 25.93 | 28.43 | 29.06 | 27.81 | 76.71 | 78.6 | 78.36 | 77.89 |
| 119 | NCS-S-1 x P/1-1 | 24.94 | 28.07 | 29.44 | 27.48 | 80.33 | 80.19 | 80.85 | 80.46 |
| 120 | Mutant 189/75 | 26.09 | 27.75 | 28.82 | 27.55 | 80.23 | 81.06 | 81.35 | 80.88 |
| 121 | N-69 | 25.69 | 26.4 | 27.99 | 26.69 | 79.58 | 80.36 | 80.62 | 80.18 |
| 122 | HG-1 | 25.79 | 28.74 | 29.42 | 27.98 | 79.38 | 80.48 | 81.22 | 80.36 |
| 123 | MAR 444-2-70 | 28.54 | 29.64 | 30.24 | 29.47 | 80.88 | 80.27 | 81.5 | 80.88 |
| 124 | Huma-15 | 23.9 | 27.33 | 28.99 | 26.74 | 78.33 | 79.13 | 78.46 | 78.64 |
| 125 | HA-7 | 25.99 | 29.2 | 29.92 | 28.37 | 79.13 | 79.86 | 80.52 | 79.83 |
| 126 | H-SU-chow | 22.03 | 28.5 | 29.04 | 26.52 | 77.83 | 79.86 | 80.49 | 79.39 |
| 127 | H 2918-2 | 25.89 | 27.79 | 28.52 | 27.4 | 78.63 | 79.39 | 80.67 | 79.56 |
| 128 | Dos-056 | 23.45 | 26.64 | 28.14 | 26.08 | 78.63 | 78.11 | 79.31 | 78.68 |
| 129 | DPL-16 | 22.89 | 28.7 | 29.14 | 26.91 | 78.88 | 79.58 | 80.58 | 79.68 |
| 130 | Delcot2775 | 23.74 | 28.6 | 28.52 | 26.95 | 78.83 | 79.86 | 79.92 | 79.53 |
| 131 | Delfos 6-343-6 | 26.3 | 28.72 | 29.69 | 28.24 | 79.73 | 79.28 | 78.84 | 79.28 |
| 132 | DPL-SR-5 | 27.19 | 29.02 | 29.54 | 28.58 | 79.78 | 80.15 | 80.5 | 80.14 |
| 133 | DT-Webber | 25.44 | 28.22 | 28.98 | 27.55 | 80.89 | 81.26 | 79.89 | 80.68 |
| 134 | Coker 310(Fg) | 25.1 | 27.9 | 28.59 | 27.2 | 78.43 | 79.41 | 79.18 | 79.01 |
| 135 | Chung Mian | 25.69 | 27 | 27.33 | 26.67 | 79.13 | 80 | 78.92 | 79.35 |
| 136 | Co2-P1 | 22.09 | 28.8 | 29.18 | 26.69 | 77.58 | 78.57 | 80.27 | 78.81 |
| 137 | Cedix | 24.55 | 27.91 | 28.73 | 27.06 | 77.58 | 78.91 | 79.41 | 78.63 |
| 138 | Acala 1517 E | 25.24 | 29 | 28.72 | 27.65 | 79.38 | 80.26 | 81.17 | 80.27 |
| 139 | A33-57 | 21.74 | 26.2 | 27.32 | 25.09 | 77.08 | 78.41 | 80.24 | 78.57 |
| 140 | PGMB-3300 | 29.53 | 29.62 | 30.08 | 29.74 | 83.96 | 80.84 | 81.11 | 81.97 |
| 141 | PGMB-1779 | 28.57 | 30.01 | 30.26 | 29.61 | 81.13 | 80.31 | 80.78 | 80.74 |
| 142 | PGMB-1408 | 30.09 | 30 | 31.1 | 30.4 | 81.86 | 81.35 | 82.37 | 81.86 |
| 143 | PGMB-3100 | 25.33 | 26.28 | 27.89 | 26.5 | 81.23 | 81.25 | 82.13 | 81.53 |
| 144 | IR-NIBGE-3701 | 30.27 | 28.1 | 30.11 | 29.49 | 81.6 | 80.52 | 81.18 | 81.1 |
| 145 | IR-NIBGE-3 | 29.89 | 29.65 | 29.11 | 29.55 | 82.39 | 81.39 | 82.34 | 82.04 |
| 146 | IR-NIBGE-901 | 30.75 | 29.85 | 30.14 | 30.25 | 82.37 | 81.39 | 80.17 | 81.31 |
| 147 | PGMB-1523 | 27.93 | 29.93 | 29.79 | 29.21 | 81.39 | 80.76 | 81.52 | 81.22 |
| 148 | PGMB-1526 | 29.47 | 28.6 | 29.38 | 29.15 | 81.73 | 81.09 | 81.46 | 81.43 |
| 149 | GN-31 | 28.77 | 27.69 | 29.22 | 28.56 | 81.11 | 81.47 | 81.98 | 81.52 |
| 150 | GN-2085 | 29.61 | 30.66 | 30.1 | 30.12 | 82.35 | 80.84 | 81.44 | 81.54 |
| 151 | AA-802 | 29.5 | 26.78 | 28.36 | 28.21 | 81.41 | 81.46 | 83 | 81.96 |
| 152 | CEMB-1 | 27.28 | 29.13 | 30.1 | 28.83 | 81.73 | 81.23 | 81.45 | 81.47 |
| 153 | IR-NIBGE-1524 | 29.43 | 28.98 | 30.13 | 29.51 | 82.15 | 81.59 | 82.31 | 82.01 |
| 154 | AA-703 | 29.18 | 29.72 | 29.15 | 29.35 | 81.92 | 81.9 | 82.74 | 82.19 |
| 155 | FH-113 | 27.3 | 27.75 | 28.3 | 27.78 | 80.76 | 80.69 | 81.47 | 80.97 |
| 156 | CEMB-2 | 25.28 | 27.26 | 28.72 | 27.08 | 79.86 | 79.31 | 79.6 | 79.59 |
| 157 | Sitara-008 | 29.48 | 29.81 | 30.1 | 29.79 | 82.52 | 80.79 | 81.24 | 81.51 |
| 158 | MG-6 | 29.18 | 28.98 | 29.62 | 29.26 | 81.83 | 81.37 | 80.79 | 81.33 |
| 159 | Neelum-121 | 29.74 | 29.61 | 30.11 | 29.82 | 81.87 | 79.91 | 81.13 | 80.97 |
| 160 | Rohi | 26.63 | 27.2 | 26.97 | 26.93 | 77.10 | 78.35 | 78.54 | 78.00 |
| 161 | LDL-113 | 25.17 | 25.29 | 25.38 | 25.28 | 77.03 | 78.18 | 78.32 | 77.84 |
| 162 | No.18 | 25.71 | 26.27 | 26.06 | 26.01 | 76.86 | 78.01 | 78.15 | 77.67 |
| 163 | FDH-113 | 26.53 | 27.04 | 26.73 | 26.77 | 77.52 | 78.68 | 78.81 | 78.34 |
| 164 | No-36 | 26.05 | 26.74 | 26.66 | 26.48 | 77.97 | 79.13 | 79.26 | 78.79 |
| 165 | No-29 | 27.07 | 27.08 | 27.3 | 27.15 | 77.94 | 79.10 | 79.23 | 78.76 |
| 166 | 212 | 26.72 | 27.26 | 27.09 | 27.02 | 78.2 | 79.35 | 79.49 | 79.01 |
| 167 | Barnecum | 25.92 | 26.53 | 26.35 | 26.27 | 76.86 | 78.01 | 78.15 | 77.67 |
| 168 | FDH-786 | 26.19 | 26.81 | 26.61 | 26.54 | 77.52 | 78.68 | 78.81 | 78.34 |
| 169 | 451-R | 27.33 | 26.86 | 27.32 | 27.17 | 77.37 | 78.53 | 78.66 | 78.19 |
| 170 | 23718 | 27.02 | 27.27 | 27.14 | 27.14 | 77.9 | 79.05 | 79.19 | 78.71 |
| 171 | M-11 | 26.84 | 27.02 | 27.14 | 27 | 78.1 | 79.26 | 79.39 | 78.92 |
| 172 | Garohill | 26.63 | 26.49 | 26.72 | 26.61 | 76.41 | 77.56 | 77.7 | 77.22 |
| 173 | D-9 | 26.19 | 26.36 | 26.42 | 26.32 | 76.55 | 77.7 | 77.84 | 77.36 |
| 174 | HK-244 | 26.73 | 26.84 | 26.74 | 26.77 | 77.44 | 78.6 | 78.73 | 78.26 |
| 175 | M-17 | 27.29 | 27.28 | 27.42 | 27.33 | 77.2 | 78.36 | 78.49 | 78.02 |
| 176 | Synthetic | 26.55 | 26.34 | 26.6 | 26.49 | 76.73 | 77.88 | 78.02 | 77.54 |
| 177 | FDH-228 | 26.1 | 26.11 | 26.36 | 26.19 | 76.81 | 77.96 | 78.1 | 77.62 |
| 178 | No-17 | 26.73 | 27.05 | 27.02 | 26.93 | 78.05 | 79.21 | 79.34 | 78.87 |
| 179 | Haroonabad | 27.31 | 27.23 | 27.38 | 27.31 | 77.08 | 78.24 | 78.37 | 77.9 |
| 180 | 27518 | 27.09 | 27.74 | 27.48 | 27.44 | 76.83 | 77.99 | 78.12 | 77.65 |
| 181 | 786 | 27.1 | 27.22 | 27.33 | 27.22 | 77.19 | 78.35 | 78.48 | 78.01 |
| 182 | 231-R | 26.15 | 26.6 | 26.62 | 26.46 | 77.23 | 78.38 | 78.52 | 78.04 |
| 183 | 450-R | 27.41 | 26.96 | 27.24 | 27.2 | 77.47 | 78.62 | 78.76 | 78.28 |
| 184 | DC-116 | 26.04 | 26.44 | 26.35 | 26.28 | 76.63 | 77.78 | 77.92 | 77.44 |
| 185 | No-35 | 26.66 | 27.25 | 27.16 | 27.02 | 78.1 | 79.26 | 79.39 | 78.92 |
| Mean | | 25.59 | 28.59 | 28.84 | 27.67 | 78.98 | 79.59 | 80.4 | 79.66 |
| Minimum | | 20.98 | 25.1 | 25.38 | 25 | 75.76 | 76.92 | 77.16 | 77.22 |
| Maximum | | 30.75 | 31.93 | 31.24 | 30.54 | 83.96 | 82.81 | 84.07 | 82.74 |
| S.D | | 1.79 | 2.89 | 2.79 | 2.49 | 2.21 | 0.55 | 1.22 | 1.33 |
| S.E | | 0.13 | 0.21 | 0.2 | 0.18 | 0.16 | 0.04 | 0.09 | 0.10 |
| C.V | | 0.07 | 0.101 | 0.097 | 0.09 | 0.028 | 0.007 | 0.015 | 0.017 |
| C.D _0.05_ | | 0.722 | 0.729 | 0.705 | 5.468 | 2.278 | 2.709 | 2.105 | 2.692 |

FSD=Faisalabad, VH=Vehari, MLN=Multan, S.D= Standard deviation, S.E=Standard error, C.V=Coefficient of variation, C.D=Critical difference

Supplementary Table 5 Principal component analysis of traits associated with 185 cotton genotypes showing eigen values and proportion variation associated with first five PC axis and eigen vectors of average boll weight, GOT percentage, micronaire value, staple length, fiber bundle strength and uniformity index

| Principal Component Axis | | | | | |
| --- | --- | --- | --- | --- | --- |
|  | 1 | 2 | 3 | 4 | 5 |
| Eigenvalues | 3.085632 | 1.124771 | 0.821783 | 0.514687 | 0.271824 |
| Cumulative Variation (%) | 51.4272 | 70.1734 | 83.8698 | 92.4479 | 96.9783 |
| Characters | Eigenvectors | | | | |
| Average boll weight | -0.681992 | -0.228785 | -0.449305 | -0.524103 | 0.072385 |
| GOT | -0.824850 | -0.141403 | -0.255423 | 0.406834 | 0.102028 |
| Micronaire value | 0.257767 | 0.771544 | -0.566645 | 0.095035 | 0.046895 |
| Staple length | -0.897381 | -0.194336 | -0.034769 | 0.207824 | 0.019321 |
| Fiber bundle strength | -0.638176 | 0.519476 | 0.464052 | -0.132731 | 0.298915 |
| Uniformity index | -0.813103 | 0.386693 | 0.130488 | -0.068180 | -0.405280 |

**Supplimentary Figures**

**
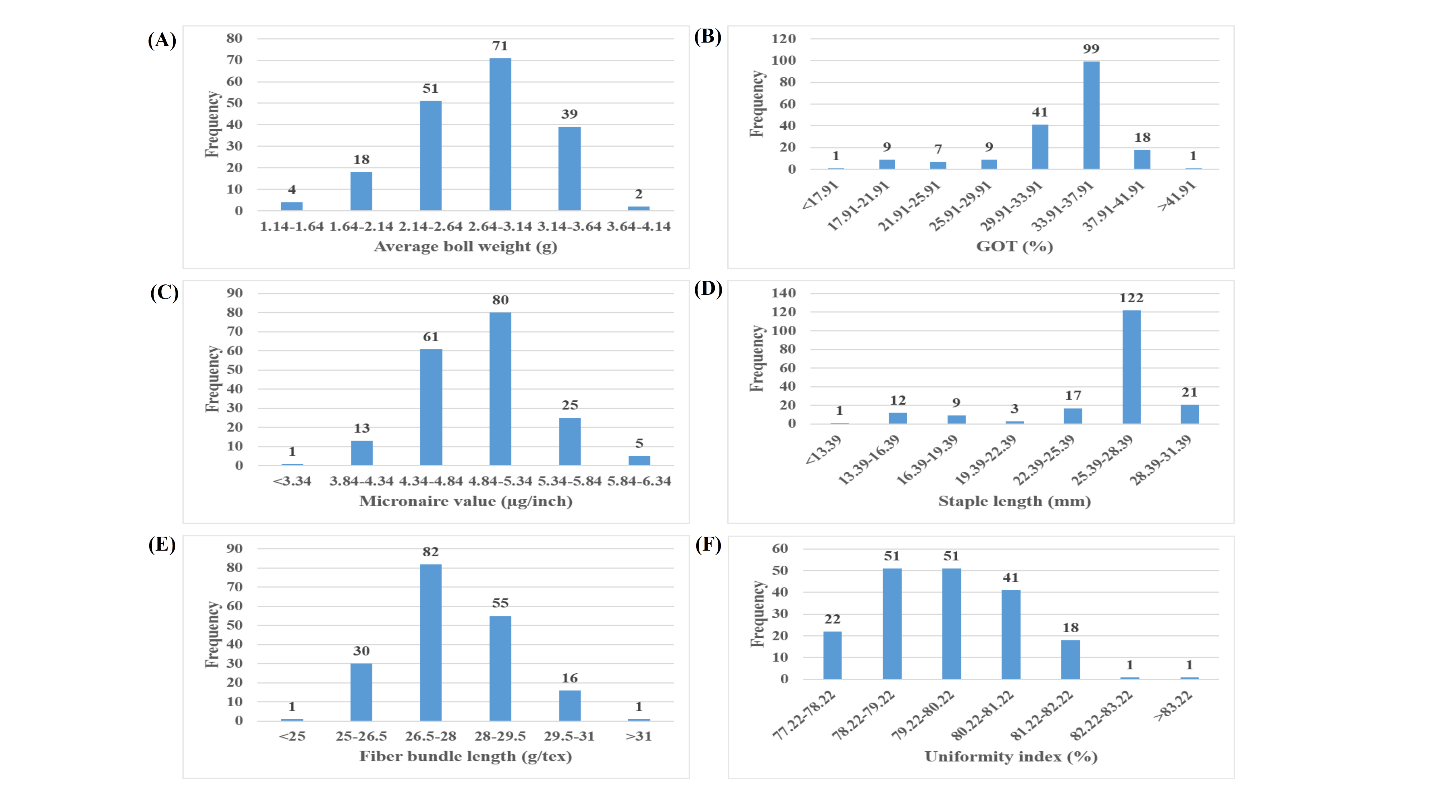
**

Supplementary Figure 1 Frequency distribution of average boll weight (A), GOT percentage (B), Micronaire value (C), Staple length (D), Fiber bundle strength (E) and uniformity index (F) of lint samples collected from NIBGE Faisalabad, CRS Vehari and CCRI Multan from 2011-13

**
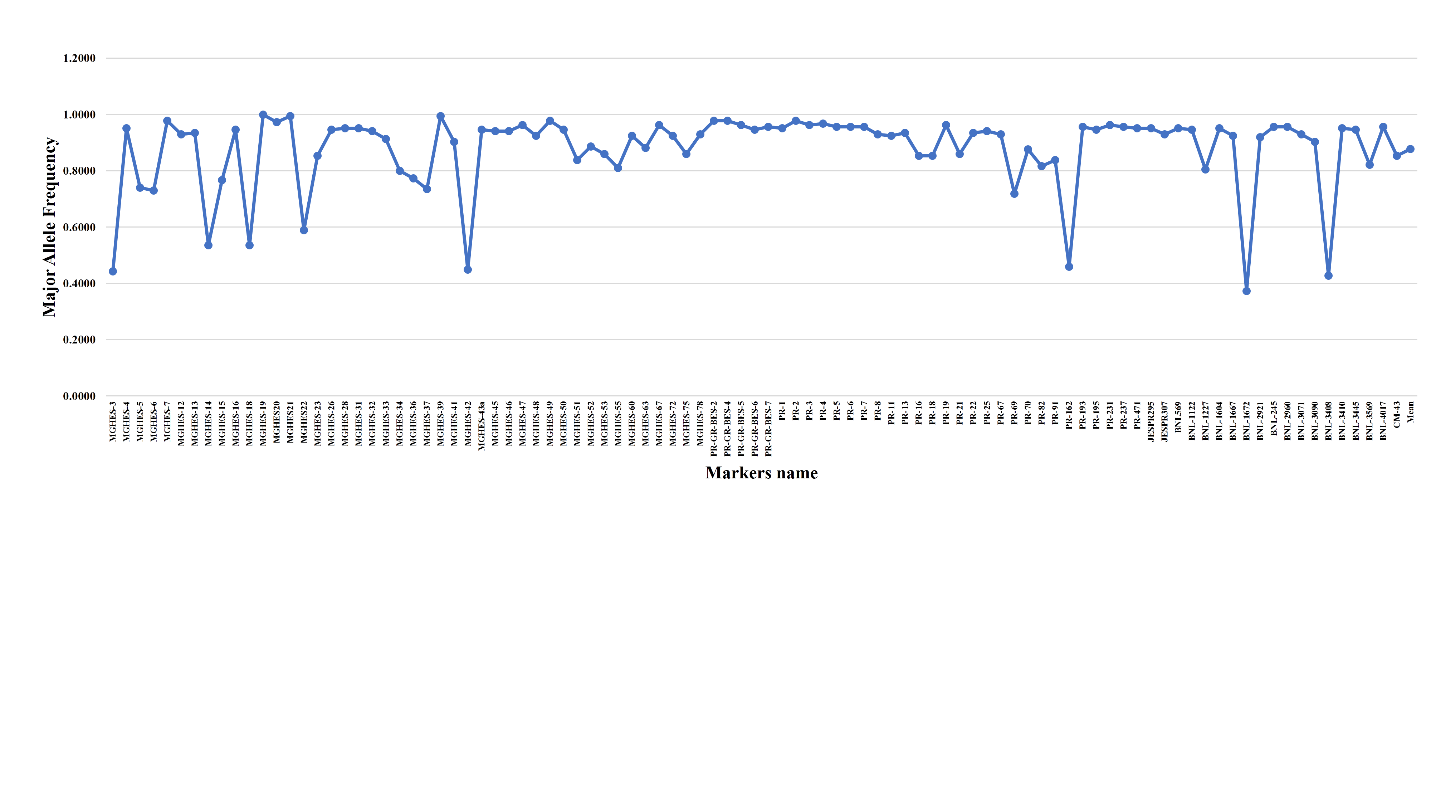
**

Supplementary Figure 2 Histogram showing the major allele frequency of polymorphic primers


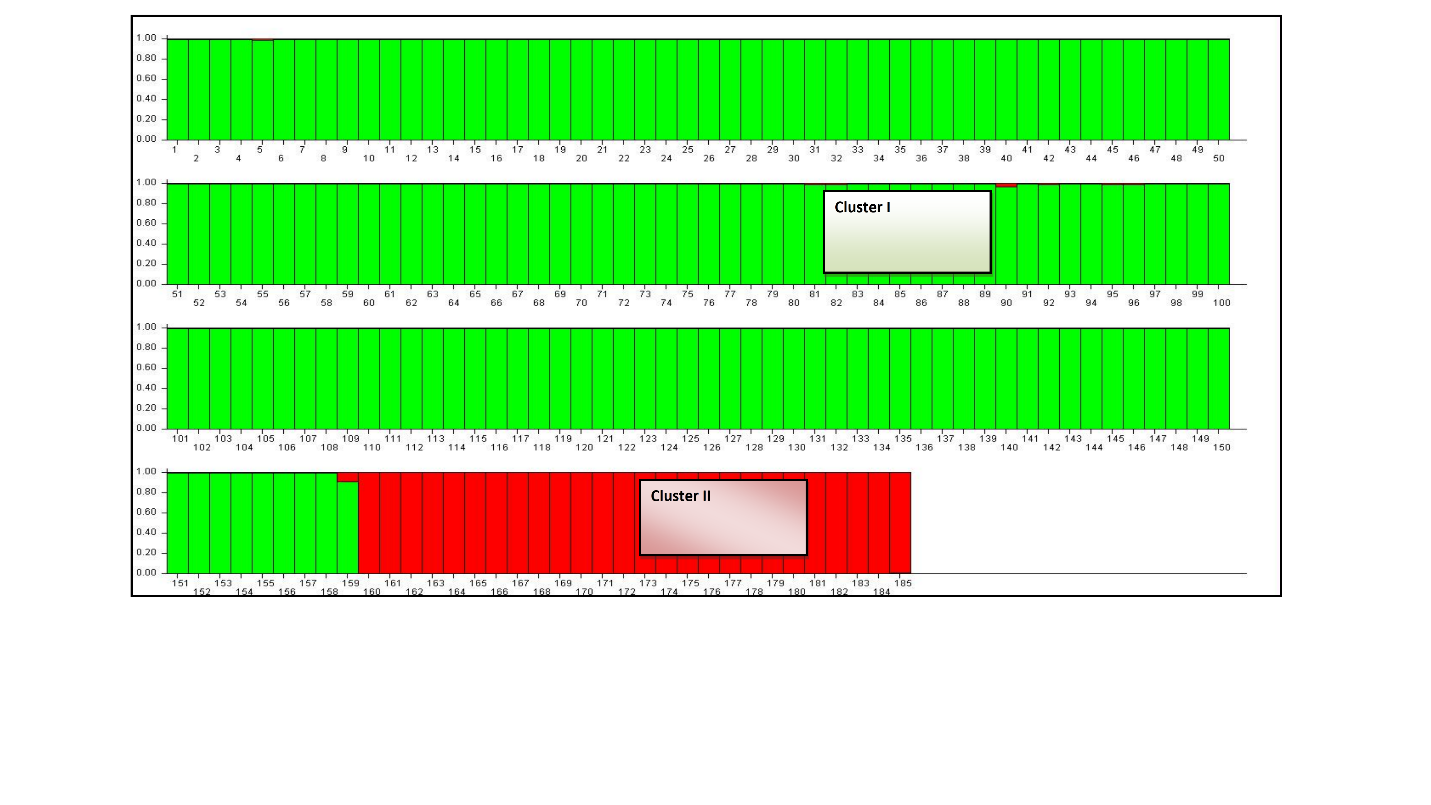


Supplementary Figure 3 The summary plot of Q-matrix estimates: cluster 1- *G. hirsutum* germplasm group (defined with the green colour); cluster 2- *G. arboreum* germplasm group (defined with the red colour)
